# TADsplimer reveals splits and mergers of topologically associating domains for epigenetic regulation of transcription

**DOI:** 10.1186/s13059-020-01992-7

**Published:** 2020-04-02

**Authors:** Guangyu Wang, Qingshu Meng, Bo Xia, Shuo Zhang, Jie Lv, Dongyu Zhao, Yanqiang Li, Xin Wang, Lili Zhang, John P. Cooke, Qi Cao, Kaifu Chen

**Affiliations:** 1 Center for Bioinformatics and Computational Biology, Houston Methodist Research Institute, Houston, TX 77030 USA; 2 Center for Cardiovascular Regeneration, Houston Methodist Research Institute, Houston, TX 77030 USA; 3 Department of Cardiothoracic Surgery, Weill Cornell Medicine, Cornell University, New York, NY 10065 USA; 4 Department of Urology, Northwestern University Feinberg School of Medicine, Chicago, IL 60611 USA; 5 Robert H. Lurie Comprehensive Cancer Center, Northwestern University Feinberg School of Medicine, Chicago, IL 60611 USA

**Keywords:** Hi-C, Topologically associating domains, Chromatin conformation, Histone modification, Bioinformatics, Computational biology, Epigenomics

## Abstract

We present TADsplimer, the first computational tool to systematically detect topologically associating domain (TAD) splits and mergers across the genome between Hi-C samples. TADsplimer recaptures splits and mergers of TADs with high accuracy in simulation analyses and defines hundreds of TAD splits and mergers between pairs of different cell types, such as endothelial cells and fibroblasts. Our work reveals a key role for TAD remodeling in epigenetic regulation of transcription and delivers the first tool for the community to perform dynamic analysis of TAD splits and mergers in numerous biological and disease models.

## Background

The three-dimensional (3D) structure of chromosomes inside the nucleus of a cell plays essential roles in the regulation of transcription, replication, and many other biological procedures [1–3]. Techniques recently developed to infer the conformation of the chromatin are providing powerful methods to uncover the relationship between genome functionality and spatial organization of chromosomes [1–3]. Particularly, the Hi-C technique generates DNA sequencing data to enable a systematic detection of 3D structures across the genome [4]. One of the key findings revealed by Hi-C is that a chromosome is divided into individual topologically associating domains (TADs) [5]. A TAD represents a spatial unit with frequent interaction between DNA sequences within the unit, but with about 2-fold fewer interactions between units [4–6]. Therefore, TADs physically restrict interactions between enhancers and promoters for transcriptional regulation [4]. The boundary of TAD is found to be highly conserved between cell types and thus stable during development [6], although the density of interaction between DNA sequences within a TAD can dynamically change during cell differentiation or reprogramming [7–10]. The long-range interaction between a promoter and a regulatory element may be cell type-specific, but generally occurs within a highly conserved TAD [11]. However, little is known about the split of individual TADs or the merger of neighboring TADs on the chromatin.

There are several lines of evidence indicating that TADs serve as functional units of the chromosome. First, genes within the same TAD often display similar changes in RNA expression during cell differentiation [5]. Second, the boundaries of a TAD constrain the spread of histone modifications and lamin association; therefore, there is a strong correlation between genomic sites in their histone modifications within a highly conserved TAD [5, 12]. Third, co-regulated genes, such as protocadherin genes, tend to co-locate in the same TAD [13]. Considering that TADs represent a functional unit of the chromosome, studying the remodeling of TADs will advance our understanding of the cell type-specific gene expression regulation by chromatin state, the long-range enhancer-promoter interaction, and the dynamics of chromatin 3D structure.

Many algorithms have been developed to define TADs in a single Hi-C sample [4, 5, 14–17]. However, systematically analyzing the reorganization of TADs in response to biological stimuli remains a technical challenge. In this study, motivated by an initial observation of splits and mergers of individual TADs in fibroblast relative to endothelial cells, we developed TADsplimer, the first algorithm to systematically detect these events across the genome. We next applied the algorithm to Hi-C data from endothelial cells, fibroblasts, 8 stages of T cell differentiation, and multiple cancer cell lines, and uncovered important biological implications of TAD split and merger events in these biological contexts.

## Results

### Develop the TADsplimer algorithm to detect TAD splits and mergers in one sample relative to another sample

**Fig. 1:**
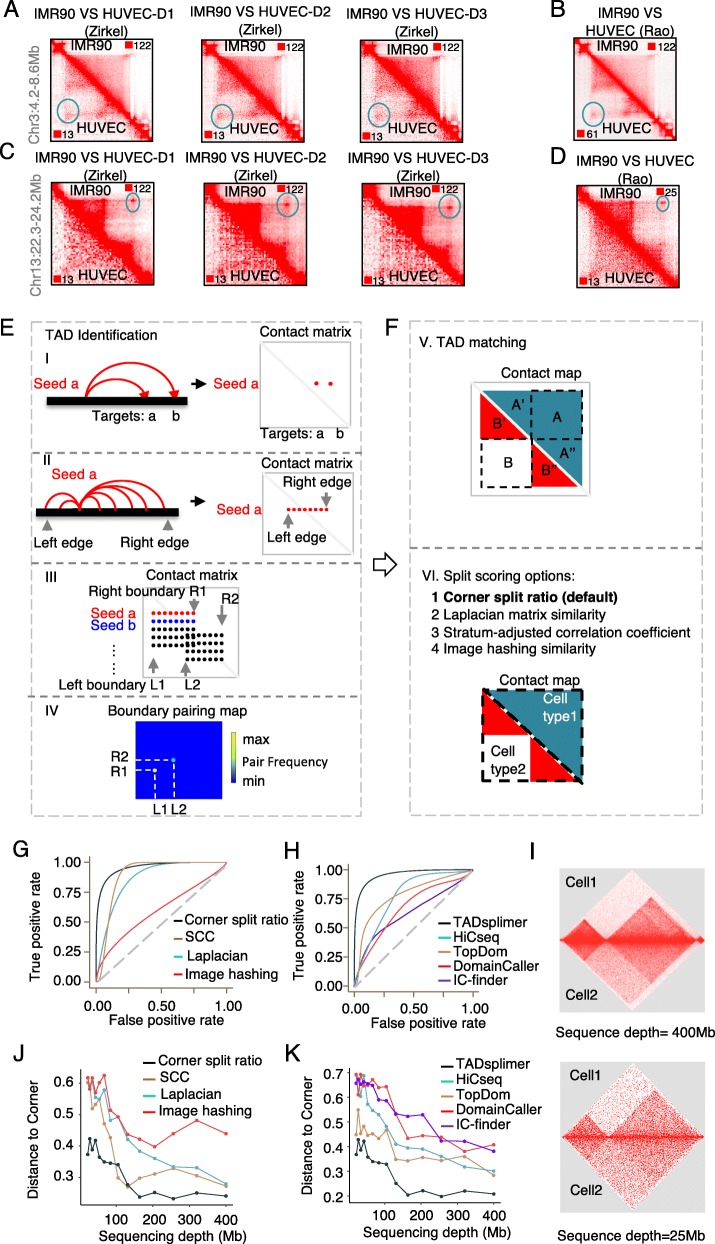
Develop the TADsplimer algorithm to detect TAD splits and mergers with high accuracy. **a**–**d** Heatmaps showing the chromatin interactions in a fibroblast TAD that was split in HUVEC (**a**, **b**) and in a HUVEC TAD that was split in fibroblast (**c**, **d**). In each heatmap, the top right triangle area indicates data for the fibroblast IMR90, and the bottom left triangle area indicates data for HUVEC. HUVEC data generated by the same lab for 3 donors was indicated by 3 heatmaps in **a** and **c**. HUVEC data generated by an additional lab was indicated in **b** and **d**. All heatmaps in **a** and **b** indicate data from the same genomic region, whereas all heatmaps in **c** and **d** indicate data from another genomic region. The blue circle indicates chromatin loops that were not disrupted by the TAD splits. Color scales for each heatmap were indicated in the top right and bottom left corners. **e** Cartoons showing steps I to IV for TAD identification in TADsplimer. **f** Cartoons showing the two steps to define TAD split or merger in one sample relative to another sample in TADsplimer. **g** ROC curve showing the performance of four alternative methods in TADsplimer for scoring TAD splits. **h** ROC curve showing the influence of five TAD identification methods on the detection of TAD splits. **i** Heatmaps showing the simulated frequency of chromatin interaction at a sequencing depth of 400 million (top) or 25 million (bottom) reads. **j** ROC curve distance to top left corner is plotted against Hi-C sequencing depth to show the performance of the four alternative methods in TADsplimer for scoring TAD splits. **k** ROC curve distance to top left corner is plotted against Hi-C sequencing depth to show the influence of the five TAD identification methods on detection of TAD splits

We initially observed that several of the TADs in human umbilical vascular endothelial cells (HUVEC) were each organized into two smaller TADs in the fibroblast (a “TAD split” in fibroblast relative to HUVEC) (Fig. 1a). This phenomenon was highly reproducible in data generated by the same lab for different donors of HUVEC (Fig. 1a) and was further observed in data generated independently by another lab for these two cell types (Fig. 1b). Similarly, we found that other genomic regions that were each defined by two neighboring TADs in the HUVEC cell were merged in the fibroblast to form a single TAD (a “TAD merger” in fibroblast relative to HUVEC) (Fig. 1c), and the phenomenon is highly reproducible in data generated by the same lab for different donors of HUVEC (Fig. 1c), and further in data generated independently by another lab for these two cell types (Fig. 1d).

To systematically investigate this phenomenon, we developed TADsplimer, the first algorithm for dynamic analysis of TAD splits and mergers based on Hi-C data from one sample relative to another sample. We designed two major functions in TADsplimer. The first function is to define individual TADs across the genome in each sample. The second function is to calculate a split score for each TAD in once sample relative to another sample. In the first function (Fig. 1e), the input data is the processed Hi-C reads that each indicates an interaction between two loci in the genome. These Hi-C reads form a contact matrix *M*, in which each element *M*_*i,j*_ is the number of reads that each indicates an interaction between the loci *L*_*i*_ and loci *L*_*j*_ (Fig. 1e, step I). For each loci *L*_*i*_, TADsplimer then defines a flanking region in which many loci interact with *L*_*i*_. This region would appear to be a strap *S*_*i*_ in the contact matrix *M*, and we will define the left and right edges for the strap (Fig. 1e, step II). If there is a TAD, the straps defined for individual loci in the same TAD will overlap with each other and thus will allow us to define TAD boundaries based on the distribution of strap edges (Fig. 1e, step III). When TADs overlap with each other, it becomes more difficult to match the two boundaries of each TAD. To solve this problem, we utilized two-dimensional kernel density estimation to calculate the probability that a pair of boundaries comes from the same TAD (Fig. 1e, step IV). In the second function, TADsplimer first matches each TAD defined in one sample to the overlapped TADs in another sample (Fig. 1f, step V). It then calculates a split score for each pair of TADs from the two samples based on the corner split ratio by default, and further allows users to calculate the split score based on three additional alternative methods, including Laplacian matrix similarity (LMS) [18], stratum-adjusted correlation coefficient (SCC) [19], and image hashing similarity (IHS) [20] (Fig. 1f, step VI).

### Simulation data demonstrated superior performance of TADsplimer

To evaluate the performance of TADsplimer, we performed a test based on a pair of simulated Hi-C datasets, in which we know the exact split or merger sites of a subset of simulated TADs. Receiver operating characteristics (ROC) curve was used to compare the methods for calculating the split score and, thus, to evaluate the accuracy for the identification of TAD splits and mergers. The AUC value for corner split ratio was 0.94, whereas the values for the SCC, LMS, and IHS methods were 0.87, 0.81, and 0.6, respectively (Fig. 1g). Therefore, the default method, corner split ratio, outperformed the three alternative methods in the detection of TAD splits and mergers.

We next determined if the methods for defining individual TADs influenced the accuracy in detecting TAD splits and mergers. We replaced our default TAD identification method with each of the four state-of-the-art methods [21], including the HiCseq [22], TopDom [16], DomainCaller [4], and IC-finder [15]. The results indicated that the default method in TADsplimer outperformed HiCseq, TopDom, DomainCaller, and IC-finder (Fig. 1h). Therefore, the method to define TAD is important to the accuracy of the detection of TAD splits and mergers, and our TAD identification method in TADsplimer has been optimal when compared to the existing methods.

Since Hi-C analysis often requires deep sequencing depth, we further investigated the influence of sequencing depth on the detection of TAD splits and mergers by TADsplimer. We down-sampled the simulated Hi-C reads (Fig. 1i) to reduce sequencing depth and evaluated the performance of each alternative method. We observed little effect on the performance of these methods when sequencing depth decreased from 400 million to 100 million, whereas all four methods for calculating split score show poorer performance when the sequencing depth continued to decrease (Fig. 1j, Additional file 1: Fig. S1A). The five methods for TAD identification also show similar sensitivity to the sequencing depth (Fig. 1k, Additional file 1: Fig. S1B). However, the default method of TADsplimer consistently displayed the best performance even after we reduced the sequencing depth to 20 million (Fig. 1j, k, Additional file 1: Fig. S1A, B). Similarly, the performance at different Hi-C resolutions is always the best for the default algorithm in TADsplimer when compared to other alternative algorithms (Additional file 1: Fig. S1C). These results again indicated that TADsplimer is an optimal algorithm for the detection of TAD splits and mergers between Hi-C samples.

### TADsplimer successfully detected TAD splits and mergers in Hi-C data from different cell types

**Fig. 2:**
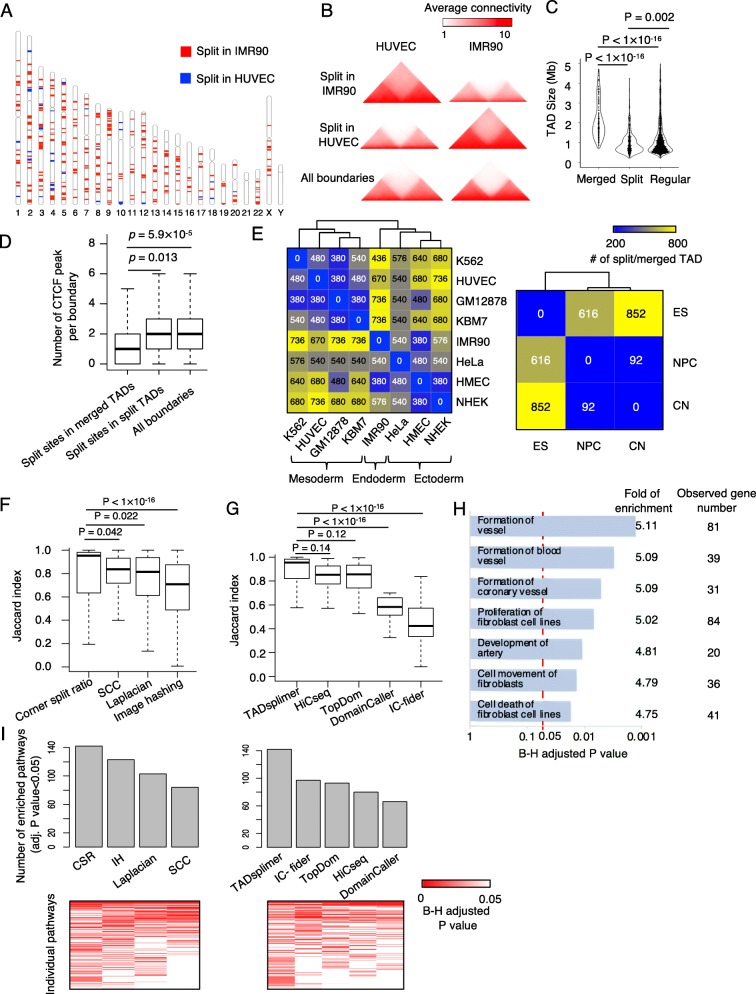
TADsplimer successfully detected TAD splits. **a** Chromosome map showing the genomic locations of fibroblast (IMR90) TADs that were split in HUVEC (blue) and HUVEC TADs that were split in IMR90 (red). **b** Heatmaps showing the average frequency of chromatin interaction in 6 aggregates: merged in HUVEC (top left) and split in IMR90 (top right), split in HUVEC (middle left) and merged in IMR90 (middle right), and all adjoint TADs in HUVEC (bottom left) and IMR90 (bottom right). **c** Violin plot of TAD sizes for merged, split, and regular TADs from HUVEC and IMR90 cells. **d** Boxplots showing the binding frequency of CTCF at the individual group of TAD boundaries. **e** Heatmaps showing the number of split TADs between cell types. **f** Boxplots showing the Jaccard index of split TADs between replicates. Results were plotted for individual TAD split identification methods. **g** Boxplots showing the Jaccard index of identified TADs between replicates. Results were plotted for individual TAD calling methods. **h** Enrichment of representative pathways in genes associated with split or merged TADs. *P* value was determined by Fisher’s exact test and adjusted by the B-H method. **i** Heatmaps and barplot showing the enriched pathways and the number of enriched pathways, respectively, for genes in split and merged TADs defined by alternative methods. *P* value was determined by Fisher’s exact test and adjusted by the B-H method

To test the performance of TADsplimer on real Hi-C data, we compared human umbilical vascular endothelial cells (HUVEC) with the fibroblast cell IMR90, as endothelial-to-mesenchymal transition and fibroblast-to-endothelial trans-differentiation are of physiological and pathobiological importance [23]. Based on a corner split ratio cutoff value of 0.45, which corresponds to a false-positive rate of 0.01, TADsplimer successfully detected 613 splits but only 67 mergers of TADs in IMR90 relative to HUVEC (Fig. 2a). The splits and mergers distributed evenly on individual chromosomes, with the number of splits plus the number of mergers ranges from 50 to 9 on each chromosome when comparing IMR90 to HUVEC. The difference in the pattern of chromatin interaction between the merged and split states of these TADs is clearly observed both by manually inspecting individual TADs (Fig. 1a) and by aligning all differential TADs around split sites to calculate average frequency (Fig. 2b). The average size of these TADs is 2.01 Mb before splitting and 0.96 Mb after splitting (Fig. 2c). Because CTCF was known to bind on TAD boundaries, we observed that the number of CTCF ChIP-Seq enrichment peaks at TAD split sites significantly increased after the splitting (Fig. 2d). We next further used TADsplimer to detect TAD splits and mergers between 8 human cell types and between 3 mouse cell types. We observed a smaller number of TAD splits and mergers between cell types that have a closer developmental relationship to each other, e.g., small number between mesoderm cell types or between ectoderm cell types and larger number between mesoderm and ectoderm cell types (Fig. 2e).

We calculated the Jaccard index to measure the reproducibility of the detected TAD splits and mergers between replicates from each of the 8 human cell types. Our results showed that the reproducibility is the highest for the default algorithm when compared to the other alternative algorithms in TADsplimer (Fig. 2f). Because we have to define TADs before defining TAD splits and mergers, we further calculated the Jaccard index to measure the reproducibility of identified TADs between Hi-C replicates from each of the 8 human cell types [12, 24]. TADsplimer showed similar performance when compared to HiCseq and TopDom and had better performance when compared to DomainCaller and IC-finder (Fig. 2g). Therefore, the default algorithm is optimal when compared to other alternative algorithms in TADsplimer for the detection of TAD splits and mergers in real Hi-C data.

We next questioned whether the TAD splits or mergers are associated with biological functions of the analyzed cell types. We retrieved genes in the TADs that were split or merged in IMR90 relative to HUVEC and submitted the genes to pathway enrichment analysis using Ingenuity Pathways Analysis (IPA, Ingenuity System Inc., USA). Fibroblast and endothelial pathways displayed significant enrichment in these genes (Fig. 2h), e.g., pathways of vessel formation, which is a major function of endothelial cells, and other pathways such as the fibroblast cell proliferation, fibroblast movement, and cell death of fibroblast. Therefore, genes in the TADs that were split or merged are enriched in the pathways important for the underlying cell types.

We further evaluated the performance of alternative algorithms in TADsplimer by comparing the enrichment of individual biological pathways in the genes associated with the detected TAD splits and mergers. For the pathways associated with TAD splits or mergers defined by at least one of the four methods for scoring TAD split, the largest number of enriched pathways was from the corner split ratio method when compared to the SCC, Laplacian, or image hashing methods (Fig. 2i). We also evaluated the influence of TAD identification methods on the enrichment of these pathways. For the pathways associated with TAD splits or mergers defined based on at least one of the five methods for TAD calling, the largest number of enriched pathways was from TADsplimer when compared to the TopDom, HiCseq, DomainCaller, or IC-finder (Fig. 2i). Therefore, TADsplimer is optimal for detecting functionally meaningful TAD splits or mergers on the base of real Hi-C data.

### Most TAD splits and mergers are independent of genetic alternations

It has been reported that genetic alterations can disrupt TADs and form neo-TADs [25, 26]. Accordingly, we investigated whether the TAD splits and mergers detected by TADsplimer are a result of genetic structure variations such as chromatin recombination, DNA deletion, duplication, or translocation. Intriguingly, although a split is associated with the loss of most interactions between DNA sequences from the two sides of a split site, chromatin loops between the two sides can remain unaltered (Fig. 1a–d). This suggested that the two TADs resulting from each of these TAD splits are still connected; therefore, that TAD splits are unlikely caused by chromatin recombination. Further, the TAD splits and mergers are highly consistent when we analyze the primary cell HUVEC from four different donors (Fig. 1a–d). Considering that the four different donors are unlikely to all have genetic alterations at the same genomic location, this further suggested that TAD splits and mergers might be independent of genetic alteration.

To further exclude the possibility that TAD splits and mergers detected by TADsplimer are due to genetic changes, we analyzed data [26] from two cancer cell lines, A549 and K562. Cancer cell lines are more likely to have genetic alterations. Whole genome sequencing (WGS) data revealed no chromatin recombination in these two cell lines but detected 9789 putative genomic structure variations (SVs) of other types. Visual inspection indicated that the TAD split site could have no overlap with these SVs (Additional file 1: Fig. S2A). Further, the patterns of change caused by SVs to chromatin interaction are both expected and observed to be different from the pattern caused by a TAD split or merger in the cell line K562 in comparison with the cell line A549 (Additional file 1: Fig. S2A-D). We identified 496 TAD splits and mergers between these cells and found none of them overlapped with the sites of SVs previously defined on the basis of the expected patterns in the same Hi-C data [26] (Additional file 1: Fig. S2E). For the 9475 putative SVs detected by WGS data [26], we observed an overlap with only 363 TADs associated with splits and mergers, which are less than 394 TADs observed on average by randomizing the genomic location of SVs 1000 times (Additional file 1: Fig. S2F). Further, the SVs detected by WGS are randomly located in the TAD and thus are not enriched at the associated split sites (Additional file 1: Fig. S2G). Together, these results indicated that most (if not all) of the TAD splits and mergers detected by TADsplimer were not likely due to genetic structure variations.

### TAD splits and mergers are associated with changes in the chromatin epigenetic state

**Fig. 3:**
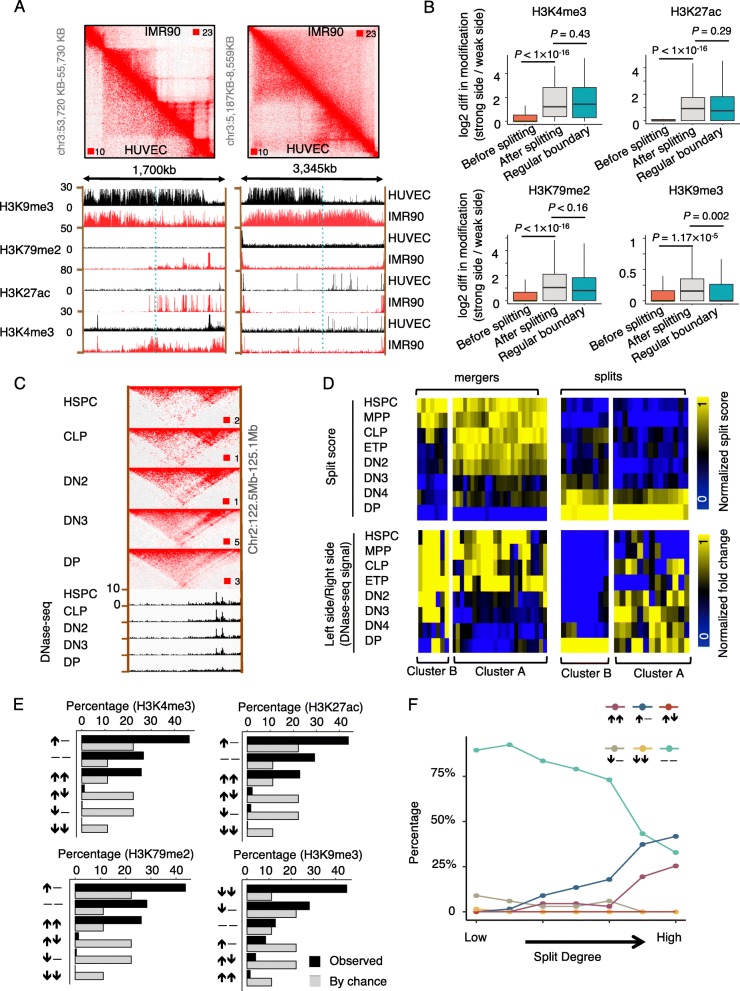
TAD splits and mergers are associated with changes in chromosome state. **a** Heatmaps of chromatin interactions determined by Hi-C (top panels) and Genome Browser tracks of ChIP-Seq signal for histone modifications (bottom panels) in HUVEC and IMR90. Vertical dash lines indicate TAD split sites. Color scales for each heatmap were indicated in the top right and bottom left corners. **b** Boxplot showing the difference in each histone modification between the two sides of TAD boundaries in HUVEC and IMR90 cells. **c** Heatmaps of chromatin interactions determined by Hi-C and DNase-Seq signal around a TAD split site at five stages of T cell lineage specification. **d** Heatmap of TAD split score (top panels) and fold difference of DNase-Seq signal between the two sides (bottom panels) of individual TAD merge sites (left panels) and split sites (right panels). **e**, **f** Percentage of TAD split sites associated with each category of histone modification change between IMR90 and HUVEC (**e**) or DNase-Seq signal change across the 8 stages of T cell lineage specification (**f**). “↑,” “↓,” and “−” denote increase, decrease, and no change of a histone modification or DNase-Seq signal at one side of the split site in response to the splitting. Each category of change is defined by changes at the two sides of the split site. For T cell lineage specification, the TAD splits are defined between DP and HSPC cells

It was reported that histone modifications are highly correlated between different sites within the same TAD, indicating coherence of chromatin state within a TAD [5, 12]. We thus investigated whether the smaller TADs derived from the split of a large TAD undergo a change in their chromatin state. We analyzed histone modifications associated with active promoter (H3K4me3), active enhancer (H3K27ac), active transcription elongation (H3K79me2), and repression of transcription (H3K9me3). We first manually inspected these histone modifications at individual TADs that were merged in HUVEC but split in IMR90 (Fig. 3a, left panels) or merged in IMR90 but split in HUVEC (Fig. 3a, right panels). We found that merged TADs tend to be in a repressed status, no matter it is merged in IMR90 or in HUVEC. Intriguingly, one of the two new TADs generated by a splitting tends to become activated, no matter the splitting happens in IMR90 or HUVEC. This observation motivated us to combine all TAD split sites from both cell types for the analysis of histone modification around the split sites in their associated cell type. In this combined analysis, if the split site is in IMR90, the histone modification data in IMR90 will be used for analysis; if the split site is in HUVEC, the histone modification data in HUVEC will be used for analysis. Similarly, we also combine all merge sites from both cell types for the analysis of histone modification around the merge sites in their associated cell type. The difference in each type of histone modification between the two sides of individual split sites is significantly larger than the difference between the two sides of individual merge sites (Fig. 3b). This observation became more obvious when the cutoff that we used to define histone modification sites was set to be more stringent (Additional file 1: Fig. S3A, B). Furthermore, we used the absolute read counts (from both peak regions and non-peak regions) at each side of the TAD split sites to perform the analysis. The result indicated that the difference in absolute read count is still bigger after splitting relative to before splitting (Additional file 1: Fig. S3C). Taking together, after a TAD splitting, the newly formed TADs at the two sides of the split site were more likely to manifest a difference in chromatin state.

We next investigated whether the TAD splits happened before or after the change in chromatin state. We therefore analyzed Hi-C and DNase-Seq data [7] to examine the dynamic interaction between the chromatin state and the split or merger of TAD at eight stages of T cell lineage specification, from the hematopoietic stem and progenitor cells (HSPCs) stage to the CD4+ CD8+ double-positive (DP) stage. TADsplimer detected 152 splits and 197 mergers of TADs in total in the DP stage when compared to the HSPC stage. Visual inspection shows that the DNase-Seq signal appears at one side of a TAD merger site before the merging and progressively decreased during the merging (Fig. 3c). For 78% of the TAD mergers, we found the mergers happened later than the change in chromatin state (Fig. 3d, left, cluster A). Similarly, for 62% of the TAD splits, the splits happened later than the change in chromatin state (Fig. 3d, right, cluster A). The rest of the TAD splits and mergers happened either earlier than or simultaneously with the change in chromatin state. Therefore, the majority of TAD splits and mergers happened after a change in chromatin state during the processes of T cell lineage specification.

We further systematically checked whether TAD splits are more likely to be associated with an increase in activating marks or repressive marks on one side of the split. We identified TADs that were merged in IMR90 and split in HUVEC, or split in HUVEC and merged in IMR90. We compared each histone modification in the merged TADs in one cell type and in their associated split TADs in the other cell type, at each side of the split site (Fig. 3e). We observed that in the majority (43–46%) of the split sites, the chromatin displayed an increase of active modifications (H3K4me3, H3K27ac, or H3K9me2) at one side and no detectable change at the other side. In another 28% of the split sites, the chromatin displayed activation as assessed by a decrease in repressive marks (H3K9me3) at one side, with no detectable change at the other side. Thus, over 65% of split sites displayed chromatin activation on at least one side of a split site, by comparing the split TADs in one cell type to their associated merged TAD in the other cell type. Intriguingly, a few split sites displayed chromatin repression (a decrease of activating modification or increase of repressing modification) at each side. We further examined the DNase-Seq data from the eight stages of T cell lineage specification (Fig. 3f). The results showed that split sites were associated with the strengthening of the DNase-Seq signal at one side and no detectable change at the other side in 41% of cases (Fig. 3f). The percentage of split sites associated with the strengthening of DNase-Seq signals at both sides increased from 0 to 25% during the splitting. In contrast, the percentage of split sites associated with no change of DNase-Seq signals at either side decreased from 88 to 33% during the splitting. Together, these data indicated that the majority of TAD splits displayed chromatin activation at one side of the split sites.

### TAD splits and mergers are associated with changes in RNA expression at one side of the split site

**Fig. 4:**
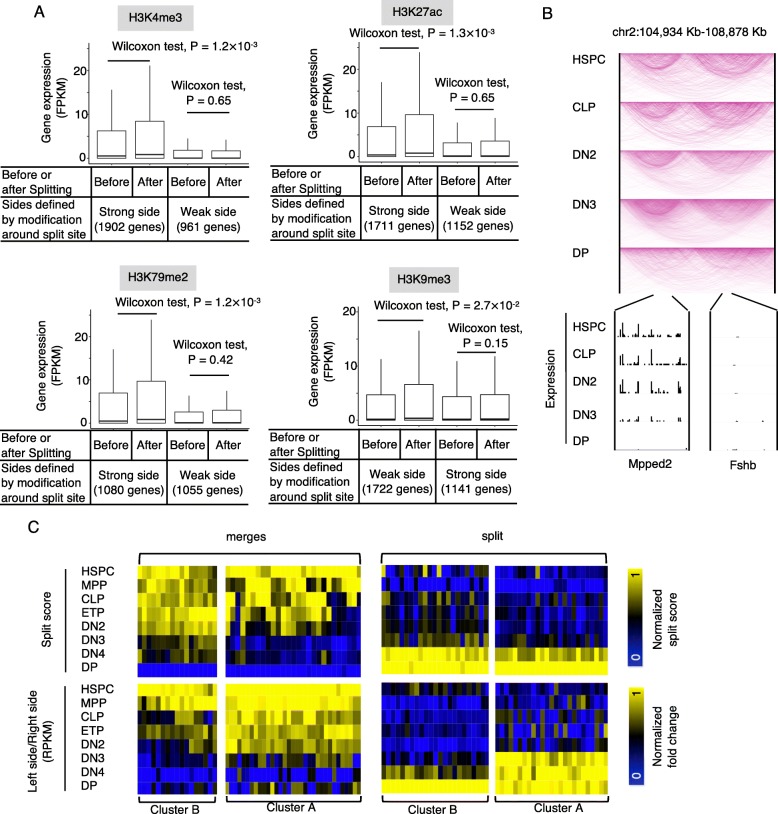
TAD splits and mergers are associated with changes in gene expression. **a** Boxplot indicating the expression level of genes at the two sides of TAD split sites before and after splitting. **b** Arc plot showing the chromatin interactions (top) and gene expression (bottom) at 5 stages of T cell lineage specification. One gene from each side of the split site was indicated. **c** Heatmaps of split scores for individual split sites (top panels) and fold difference of gene expression between the two sides of the split site (bottom panels) at 8 stages of T cell lineage specification. Genes with an expression value (FPKM) larger than 1 were analyzed

The change of chromatin state at one side of the TAD split site in response to the splitting suggests that gene expression level may also change at one side. To test this hypothesis, for each split site in IMR90, we first defined the side that displays a strong signal of a given histone modification in IMR90 and defined the other side as displaying a weak signal of that histone modification. We also defined the two sides for each split site in HUVEC based on the histone modification in HUVEC. The split sites were then combined to perform statistical analysis. We compared the expression level of genes at the strong modification side before and after TAD splitting, and further did the comparison at the weak modification side. The average number of genes in each split site is 4.2. We found that the gene expression was significantly upregulated at the active (strong active modification or weak repressive modification) side after TAD splitting, while the gene expression shows little change at the other side (Fig. 4a). This result indicated that after TAD splitting, the gene expression tends to be activated at one side of the split site and shows no detectable change at the other side.

We further used data from the 8 stages of T cell lineage commitment to investigate whether the gene expression change happens before or after TAD splits and mergers. We first analyzed merge sites between TADs that merged during T cell lineage specification, as indicated by a decrease of the split score. We observed a large difference in the expression of genes at the two sides of these sites in HSPC and a reduction in this difference during the T cell lineage specification. For example, the genes Mpped2 and Fshb are at the two sides of a TAD merge site; the gene Mpped2 was expressed in HSPC but repressed in double-positive T cells, whereas the gene Fshb was repressed at all 8 stages of T cell lineage specification (Fig. 4b). For 62% of these TAD merge sites, the mergers happened earlier than the decrease in the expression difference between the two sides (Fig. 4c, left, cluster A). For the other TAD merge sites associated with merged TADs, the mergers happen either later than or concurrently with the decrease of the expression difference between the two sides of a split site (Fig. 4c, left, cluster B). For TADs that were split during T cell lineage specification, we observed an increased difference in the expression of genes between the two sides of the split site. For 50% of these split sites, the increase of split score preceded the increase of expression difference (Fig. 4c, right, cluster A), whereas the other split sites displayed an increase of split score either later than or concurrently with the increase of the expression difference between the two sides of the split site (Fig. 4c, right, cluster B). We further assessed what percentage of changes in the gene expression can be explained by TAD split or merger. We identified 2514 genes with differential expression between HSPC and DP. About 10% (242/2514) of these genes are associated with TAD mergers or splits. Together, these results indicated that the majority of TAD splits or mergers happened earlier than gene expression changes, although some of them appear to happen concurrently with or later than expression changes.

## Discussion

Recent studies revealed that most TAD boundaries are highly conserved across cell types [12]. It is also known that changes in a few individual TADs have biological implications for cell differentiation, development, and diseases [12, 27–31]. In this study, we highlighted the phenomenon of splits and mergers of TAD regions. We developed a novel computational algorithm, TADsplimer, for detecting TAD splits and mergers based on Hi-C data from one biological sample relative to another sample. Simulation data indicated the optimal performance of the default algorithm in TADsplimer when compared to alternative algorithms. Using Hi-C data from multiple cell types, we demonstrated that TADsplimer successfully detected functionally relevant splits and mergers of TADs. TADsplimer is novel and may be of great scientific utility in the following three aspects. First, TADsplimer is the first computational tool to analyze splits and mergers of TADs between two Hi-C samples. Previous tools are designed to define individual TADs based on each single Hi-C sample. Little attention is paid to the comparison of TAD structure between different biological conditions, for example, at different stages during cell differentiation. Second, we used an integrated statistical framework to jointly optimize the performance of both TAD identification and analysis of TAD splits and mergers. This allowed TADsplimer to significantly improve the accuracy for detection of TAD splits and mergers when compared to other state-of-the-art TAD identification methods. Third, we provided a simulation data to estimate the performance of different algorithms and also used the significance of pathway enrichment to evaluate the performance based on real Hi-C data. The results demonstrated that TADsplimer successfully identified biologically significant TAD splits and mergers with high accuracy.

Recent studies have revealed that during cell differentiation, stimulation response, or cancer development, chromatin 3D structure is reorganized by A/B compartment switching events [7, 32, 33]. Chromatin 3D structure is also reported to be reorganized due to genetic alterations, e.g., DNA copy number variation and translocations [32, 34–37]. At the level of TADs, it has been reported that a subset of TADs shows changes in interaction frequency within each TAD during cell lineage specification [10]. In this study, we found that a number of TADs can change their structures by splitting or merging in one cell type relative to another cell type, somatic cell types, cancer cell lines, and individual cell types at the 8 stages of T cell differentiation. The structure changes of those TADs are associated with epigenetic alterations in the chromatin and changes of the gene expression. Pathway enrichment analysis also shows that genes in the structurally changed TADs were highly associated with the differentiation pathways of these cells.

## Conclusions

We have demonstrated that TADsplimer overcomes the unique challenges of systematically detecting the splits and merges of TADs between Hi-C samples. We further developed the first set of benchmarks to evaluate the accuracy of the identification of TAD splits and merges. Our computational pipeline, TADsplimer, will serve as a valuable tool to compare TADs under different biological conditions and facilitate the functional understanding of chromatin structure organization in numerous biological models and disease processes.

## Methods

### TAD identification

In the first step of the algorithm, we denote the Hi-C matrix as *M* = {*M*_*i*, *j*_ : *i* = 1 : *n*, *j* = 1 : *n*}, where *M*_*i,j*_ is the contact probability between bins *i* and *j*. For bin *i*, we define a contact strap *S*_*i*_ as:

1 $$ {S}_i=\left\{{M}_{i,t}:{L}_i\le t\le {R}_i\right\}, $$

where *L*_*i*_ is the left edge of the contact strap, and *R*_*i*_ is the right edge of the contact strap (Fig. 1c, II). Let *Y*_1_ and *Y*_2_ denote the random variables for the observed intra-strap contact and extra-strap contact. We model the normalized Hi-C data by a Gaussian distribution as follows:

2 $$ {Y}_1\sim N\left({\mu}_1,{\sigma}_1^2\right), $$

3 $$ {Y}_2\sim N\left({\mu}_2,{\sigma}_2^2\right), $$

where *N*(*μ*, *σ*^2^) is a Gaussian distribution with mean *μ* and variance *σ*^2^. Parameters of two Gaussian distributions are estimated using the maximum likelihood estimation. For bin *i*, we then use the binary segmentation method [38], which is widely used in the detection of change point, to estimate the position for *L*_*i*_ and *R*_*i*_. Formally, to infer the position of *L*_*i*_ and *R*_*i*_, we define the following likelihood ratio of bin *i* as a cost function which is maximized using the binary segmentation method:

4 $$ LR=L\left({M}_{i,1:{L}_i}\right)+L\left({M}_{i,{R}_i:n}\right)+L\left({M}_{i,{L}_i:{R}_i}\right)-L\left({M}_{i,1:n}\right) $$

We apply the binary segmentation method to estimate the position of the left and right edges for all straps. To calculate the distribution of the left and right edges, we estimate the density function using the Gaussian distribution as the smooth kernel function and then calculate the probability for each bin.

In the second step of the algorithm, we infer TAD boundaries based on an assumption that strap edges of a TAD are distributed around its boundary (Fig. 1c, step III). The distribution of TAD boundaries is estimated based on a Bayesian method [39] using the distribution of strap edges as priors. In this part, we summarize their main model and present the changes made to the algorithm for detecting TAD boundaries. Let ${E}^L=\left\{{E}_i^L:i=1:n\right\}$ and ${E}^R=\left\{{E}_i^R:i=1:n\right\}$ denote the possibility of the left and right edges in each bin estimated in the first step. We next estimate the distribution of the left and right boundaries. We assume that there is a partition that divides *E*^*L*^ (or *E*^*R*^) into contiguous block, such means of *E*^*L*^ (or *E*^*R*^) are equal within each block but different between neighboring blocks. The partition is initialized as a zero partition *ρ* = {*U*_1_, *U*_2_, ⋯, *U*_*n*_} = {0, 0, ⋯, 0, 1}, where *U*_*i*_ = 1 indicates a block change point at bin *i* + 1 and then updates *ρ* by a Markov chain Monte Carlo (MCMC) method. In each step of the Markov chain, a value of *U*_*i*_ is drawn from the conditional distribution of *U*_*i*_ given *E*^*L*^ (or *E*^*R*^) and the current bin *i*. From Barry et al.’s work, we know that the transition probability, *p* = {*p*_1_, *p*_2_, ⋯, *p*_*n*_}, is:

5 $$ {\displaystyle \begin{array}{c}\frac{p_i}{1-{p}_i}=\frac{P\left({U}_i=1|X,{U}_j,j\ne i\right)}{P\left({U}_i=0|X,{U}_j,j\ne i\right)}\\ {}=\frac{\int_0^{p_0}{t}^m{\left(1-t\right)}^{n-m-1} dt}{\int_0^{p_0}{t}^m{\left(1-t\right)}^{n-m} dt}\bullet \frac{\int_0^{w_0}\frac{w^{\frac{m}{2}}}{{\left({W}_1+{B}_1w\right)}^{\frac{n-1}{2}}} dw}{\int_0^{w_0}\frac{w^{\frac{m}{2}}}{{\left({W}_0+{B}_0w\right)}^{\frac{n-1}{2}}} dw},\end{array}} $$

where *X* is *P*_*L*_ or *P*_*R*_; *W*_0_, *B*_0_, *W*_1_, and *B*_1_ are the within- and between-block sums of squares obtained when *U*_*i*_ = 0 and *U*_*i*_ = 1, respectively; *m* is the number of blocks; and *p*_0_ and *w*_0_ are the hyperparameters of priors to control the sensitivity of the algorithm. Then, the posterior means are conditionally updated based on the current partition after each iteration and is used to infer the possibility of a TAD boundary at each locus. The probability density function of the left and right boundaries is:

6 $$ {f}^L(x)=\sum \limits_{i=1}^n{\mu}_i^L\bullet {1}_{B_i}(x), $$

7 $$ {f}^R(x)=\sum \limits_{i=1}^n{\mu}_i^R\bullet {1}_{B_i}(x), $$

8 $$ {1}_{B_i}(x)=\left\{\begin{array}{c}1, if\ x\in {B}_i\ \\ {}0,\mathrm{otherwise}\end{array}\right., $$

where ${\mu}_i^L$ and ${\mu}_i^R$ are the posterior means, and *B*_*i*_ is the *i*th block.

In the third step of the algorithm, we estimate the probability that a pair of the left and right boundaries forms a TAD (Fig. 1c, IV). Let $W=\left\{\left({E}_i^L,{E}_i^R\right):i=1:n\right\}$ denotes each pair of the left and right edges for each strap *S*_*i*_. We use the bivariate normal kernel to estimate the probability density function of *W* which is denoted as *f*^*P*^(*x*^*L*^, *x*^*R*^). The probability of TAD located between bin *m* and bin *n* can be written as follows:

9 $$ {P}_{m,n}^T=\sum \limits_{m\le i,j\le n}{f}^L(i)\bullet {f}^R(j)\bullet {f}^P\left(i,j\right). $$

### Differential signal calculation

Considering two Hi-C experiments under two conditions, *c*_1_ and *c*_2_, we first use a set of TADs *τ* = {*τ*_1_, *τ*_2_, ⋯, *τ*_*t*_} in the first biological condition as a reference to infer the matched TADs in the second condition. A coding tree of TADsplimer algorithm is defined as follows:

Initiation *τ* = {*τ*_1_, *τ*_2_, ⋯, *τ*_*t*_}, where *l*_*i*_ is the left boundary of *τ*_*i*_ and *r*_*i*_ is the right boundary of *τ*_*i*_, *p* = 1, *P* = {}.

Use Hi-C contact map of *c*_2_ as input to calculate *f*^*L*^, *f*^*R*^, and *f*^*P*^;

for *i* = {1, 2, ⋯, *t*},

set *l*_0_ = *l*_*i*_, *r*_0_ = *l*_*i*_;

while *l*_0_ < *r*_*i*_

Choose *l*_0_ ≤ *γ* ≤ *r*_*i*_ such that the *f*^*L*^(*l*_0_) ∙ *f*^*R*^(*γ*) ∙ *f*^*P*^(*l*_0_, *γ*) is maximized:

set *p* = *p* ∙ *f*^*L*^(*l*_0_) ∙ *f*^*R*^(*γ*) ∙ *f*^*P*^(*l*_0_, *γ*);

set *l*_0_ = *γ*;

set *p* = *p* ∙ *f*^*L*^(*l*_0_) ∙ *f*^*R*^(*r*_*i*_) ∙ *f*^*P*^(*l*_0_, *r*_*i*_);

Append *p* to *P*:

set *p* = 1;

return *P*.

Using the same strategy for two conditions, TADsplimer outputs the possibility that a big TAD matches with small TADs for each TAD in two conditions.

As TADsplimer allows hierarchical structures of TADs, it is possible that small TADs that are matched with the big TAD are sub-TADs but not truly split TADs. To avoid this problem, we further calculate four differential scores to measure the similarity of TADs under two experimental conditions. The four differential scores are corner split ratio (CSR), Laplacian matrix similarity (LMS), stratum-adjusted correlation coefficient (SCC), and image hashing similarity (IHS). To calculate the corner split ratio, let $\left({L}_{T_i}^{c_1},{R}_{T_i}^{c_1}\right)$ denotes a pair of the left and right boundaries for TAD *T*_*i*_ in the condition *c*_1_, and $\left\{\left({L}_{T_j}^{c_2},{R}_{T_j}^{c_2}\right):j=1:s\right\}$ denotes a set of pair of the left and right boundaries for TADs in condition *c*_2_ which are matched with *T*_*i*_. In conditions *c*_1_ and *c*_2_, we further denote the sub-matrix of contact probability on $\left\{\left({L}_{T_j}^{c_2},{R}_{T_j}^{c_2}\right):j=1:s\right\}$ as:

$$ {T}_S^{c_1}={\bigcup}_{j=1:s}\left\{{M}_{h,k}^{c_1}:{L}_{T_j}^{c_1}\le h,k\le {R}_{T_j}^{c_1}\right\}, $$

$$ {T}_S^{c_2}={\bigcup}_{j=1:s}\left\{{M}_{h,k}^{c_2}:{L}_{T_j}^{c_2}\le h,k\le {R}_{T_j}^{c_2}\right\}, $$

and denote the sub-matrix of contact probability on $\left({L}_{T_i}^{c_1},{R}_{T_i}^{c_1}\right)$ as:

$$ {T}_B^{c_1}=\left\{{M}_{h,k}^{c_1}:{L}_{T_i}^{c_1}\le h,k\le {R}_{T_i}^{c_1}\right\}, $$

$$ {T}_B^{c_2}=\left\{{M}_{h,k}^{c_1}:{L}_{T_i}^{c_2}\le h,k\le {R}_{T_i}^{c_2}\right\}. $$

We estimate the mean of ${T}_S^{c_1}$, ${T}_S^{c_2}$, ${T}_B^{c_1}\cap \overline{T_S^{c_1}}$, and ${T}_B^{c_2}\cap \overline{T_S^{c_2}}$ as ${\mu}_S^{c_1}$, ${\mu}_S^{c_2}$, ${\mu}_K^{c_1}$, and ${\mu}_K^{c_2}$, respectively. The corner split ratio is defined as:

10 $$ \rho \left({T}_B^{c_1},{T}_B^{c_2}\right)=\left\Vert \frac{\mu_K^{c_1}}{\mu_S^{c_1}}-\frac{\mu_K^{c_2}}{\mu_S^{c_2}}\right\Vert, $$

where || || is the Euclidean distance.

Three alternative differential score methods are Laplacian matrix similarity (LMS), stratum-adjusted correlation coefficient (SCC), and image hashing similarity (IHS). We use the HiC-spector, the HiCRep package, and the OpenCV to apply LMS, SCC, and IHS for measuring the similarity of each TAD between the two conditions.

### Simulation of Hi-C data

We first simulate TAD boundaries for two conditions. We generate Hi-C contact matrix that contains 20 TADs for each condition, with a bin size of 10 kb in the matrix. For one condition, we simulate 20 TADs of which the size ranges from 100 to 500 bins. For the other condition, we randomly select 5 TADs to merge with the TAD next to them. Second, we simulate the contact probability for two conditions based on a Poisson distribution. Considering the effect of the distance [40], we simulate the contact probability of each TAD between bin *i* and bin *j* as:

11 $$ \log \left({M}_{i,j}\right)=\mathrm{Poisson}\left(\frac{\mu }{\left|i-j\right|}\right), $$

where *μ* is the mean of contact probability in each TAD which is set to range from 1 to 3. To estimate the performances of prediction on simulation data, we apply the ROC curve using the ROCR package.

### Processing of Hi-C data

All Hi-C data were processed using the bioinformatics toolkit Juicer version 1.13.01 (https://github.com/aidenlab/juicer/wiki) [41]. We used the Juicer with default parameters to map human Hi-C reads against the reference genome version hg19 to generate the .hic file, which is a compressed binary file that contains contact matrices, Similarly, mouse Hi-C reads were aligned against the reference genome version mm9 to generate the .hic files using Juicer with default parameters. We then used the Dump function in Juicer to extract .txt files, the matrix format of contact matrices, from the .hic files at a resolution of 10 kb with vanilla coverage normalization [12]. Vanilla coverage normalization is a method in which the value of each element in a matrix is first divided by the sum of all values in the associated row and subsequently further divided by the sum of all values in the associated column.

### Visualization of Hi-C data

Heatmaps of Hi-C matrices were visualized using the bioinformatics tool Juicebox (https://aidenlab.org/software.html) [42] at a resolution of 10 kb using “coverage” normalization method, which applies vanilla coverage normalization to the Hi-C matrices. The color in each heatmap indicates the normalized number of Hi-C reads. Arc plot of Hi-C data was generated using the WashU Epigenome Browser (https://epigenomegateway.wustl.edu).

### Identification of TAD split/mergers

A full tab-separated value (TSV) matrix at a resolution of 10 kb is used as the input for TADsplimer. In the real Hi-C data analysis of our study, this input data is the output contact map from the tool Juicer, with vanilla coverage normalization at 10 kb resolution. The row and column of the input matrices represent two genomic loci, and the value of an element in the input matrices indicates the frequency of interaction between the two genomic loci associated with the row and column. We used the corner split ratio method in TADsplimer to identify TAD splits or mergers with a cutoff value of 0.45, which corresponds to a false-positive rate (FDR) of 0.01. All split scores used for further analysis were calculated by the corner split ratio method described above in detail. We used the *z*-score method to further normalize the split score for comparison between the eight stages of T cell differentiation (Figs. 3d and 4c).

### Assessing the performance of TADsplimer

To define TADs, we used four existing tools, including HiCseq, TopDom, DomainCaller, and IC-finder. For HiCseq, we used the Gaussian distribution method to identify TADs. The other parameters of HiCseq were set as the default values. For TopDom, the parameter window size was set to 10. The other parameters were set as the default values. For IC-Finder, all parameters were set as default values. For DomainCaller, we used the window size 500 kb to correspond to the window size setting described in the main document for the 10 kb. To calculate the TAD split score, we used four methods including CSR, LMS, SCC, and IHS. A FDR of 0.01 was used as the cutoff for each method. To measure the performance of TAD identification in real Hi-C data, we calculate the Jaccard index for the overlap of the identified TADs between replicates. The Jaccard index was defined as the number of overlapped TADs (intersection) divided by the number of all TADs (union) from both replicates [43]:

$$ J\left({W}_1,{W}_2\right)=\frac{\mid {W}_1\cap {W}_2\mid }{\mid {W}_1\cup {W}_2\mid }, $$

where *W*_1_ and *W*_2_ are the length TADs. The performance of TAD splits and mergers identified in real Hi-C data was also measured by the Jaccard index of overlap between replicates.

### Analysis of ChIP-Seq data

For ChIP-Seq data analysis, the sequencing reads from human cell lines were mapped to the human reference genome version hg19, and the sequencing reads of mouse cell lines were mapped to the mouse reference genome version mm9 using the bioinformatics tool Bowtie version 1.1.0 with default parameters. Only the uniquely mapped reads were used for downstream analysis. We used the Danpos version 2.2.2 [44, 45] to define ChIP-Seq or DNase-Seq enrichment peaks. We normalized the average reads density across the genome to 0.65 reads/bp using Danpos2. The “Dpeak” function in Danpos2 was used for peak calling with default parameters. In the read data analysis, the signal of ChIP-Seq data is defined as the normalized ChIP-Seq read count at each base pair.

### Analysis of RNA-Seq data

The reference gene set UCSC Known Genes were downloaded from the UCSC Genome Browser website (http://hgdownload.soe.ucsc.edu/downloads.html) [46]. We mapped the RNA-Seq reads to the human genome version hg19 or mouse genome version mm9 using TopHat (v2.0.12) with default parameters. The bioinformatics tool Cuffdiff (version 2.0.12) was used to calculate the gene expression level and significance of differential expression based on the classic-FPKM with default parameters. To analyze the changes of RNA expression in the split or merged TADs between the eight stages of T cell differentiation, we first calculated the fold difference of FPKM value for each possible pair of genes from the two sides of each split site. We then analyzed whether this fold difference increases or decreases in response to TAD splits or mergers. Expressed genes, whose FPKM values are larger than 1, were included in the analysis.

### Pathway enrichment analyses

Ingenuity Pathway Analysis (IPA) for genes that are located in the split or merged TADs was performed. Both the canonic pathways and functional pathways in the annotation database of IPA were used to do the pathway enrichment analysis. The IPA used Fisher’s exact test to determine the significance of enrichment and used the Benjamini-Hochberg (B-H) methods to adjusted the *p* value for multiple test. A B-H-adjusted *p* value cutoff of 0.05 was used to select significantly enriched pathways. Given that there are *n*, *n*_1_, and *n*_2_ genes in the reference gene set, in the split and merged TADs, and in an IPA pathway, the number of overlap expected by random chance between the *n*_1_ genes and the *n*_2_ genes will be *n*_*e*_ = *n*_1_ × *n*_2_/*n*. Given that the observed number of overlap between the *n*_1_ genes and the *n*_2_ genes is *n*_*o*_, then the fold enrichment for this pathway will be *f* = *n*_*o*_/*n*_*e*_. The IPA has a function to perform a comparative analysis of pathway enrichment between multiple different gene sets; we therefore used this function to compare between genes in the split and merged TADs defined by the multiple alternative algorithms in TADsplimer.

### Statistical analyses

All statistical analyses were conducted using the R version 3.5.1. Wilcoxon test was performed using the function wilcox.test from the stats package. The permutation test was performed using the permTS function from the perm package (https://cran.r-project.org/web/packages/perm/index.html) with default parameters. The circular permutation test was performed using the the enrichmentAnalysis function from the shiftR package (https://cran.r-project.org/web/packages/shiftR/index.html) with a npermute parameter value of 1000.

## Supplementary information

**Additional file 1: Supplementary Figure S1-S3.**

**Additional file 2:** Review history.

## Data Availability

The Hi-C data for the human cell lines IMR90, HUVEC, K562, GM12878, KBM7, Hela, HMEC, and NHEK were downloaded from the GEO database with the accession number GSE63525 [12]. The Hi-C data for HUVEC were downloaded from the GEO database with the accession numbers GSE63525 [12] and GSM2595581 [27]. The Hi-C data for the eight stages of T cell differentiation and the cell line A549 were downloaded from the GEO database with the accession numbers GSE79422 [7] and GSM2437834, respectively. The Hi-C data for mouse ES, NPC, and CN were downloaded from the GEO database with the accession number GSE96107 [24]. The RNA-Seq data and ChIP-Seq data for IMR90 and HUVEC were downloaded from the ENCODE project website (https://www.encodeproject.org/). Specifically, the accession numbers for H3K79me2, H3K27ac, H3K4me3, and H3K9me3 from the IMR90 cell line are ENCSR831JSP, ENCSR002YRE, ENCSR087PFU, and ENCSR055ZZY, respectively. The accession numbers for H3K79me2, H3K27ac, H3K4me3, and H3K9me3 from the HUVEC cell are ENCSR000ASD, ENCSR000ALB, ENCSR578QSO, and ENCSR000ATB, respectively. The accession numbers for RNA-Seq data from the IMR90 and HUVEC cells are ENCSR000CTK and ENCSR000COZ, respectively. The RNA-Seq data and DNase-Seq data for the eight stages of T cell differentiation were downloaded from the GEO database with the accession number GSE79422 [7]. WGS data for A549 and K562 were downloaded from the SRA database with the accession number PRJNA380394 [26]. We downloaded the genome location of SVs that were detected by WGS and Hi-C data in A546 and K562 cell lines from a recent publication [26]. A python package implementing the TADsplimer algorithm is available at the following website: https://github.com/GuangyWang/TADsplimer.git [47]. DOIs for the individual releases of TADsplimer are available under DOI: 10.5281/zenodo.3553025 [48]. The source code is released under an open-source license compliant with the MIT License which is approved by Open-Source Licenses (OSI) (https://opensource.org/licenses).
