# Supplementary material for: TADsplimer reveals splits and mergers of topologically associating domains for epigenetic regulation of transcription
Source: Genome Biol. 2020 Apr 2;21:84. doi: 10.1186/s13059-020-01992-7 (PMC7114812; doi:10.1186/s13059-020-01992-7)
Supplement: Supplementary file 1 — Additional file 1: Supplementary Figure S1-S3. [file 13059_2020_1992_MOESM1_ESM.docx]

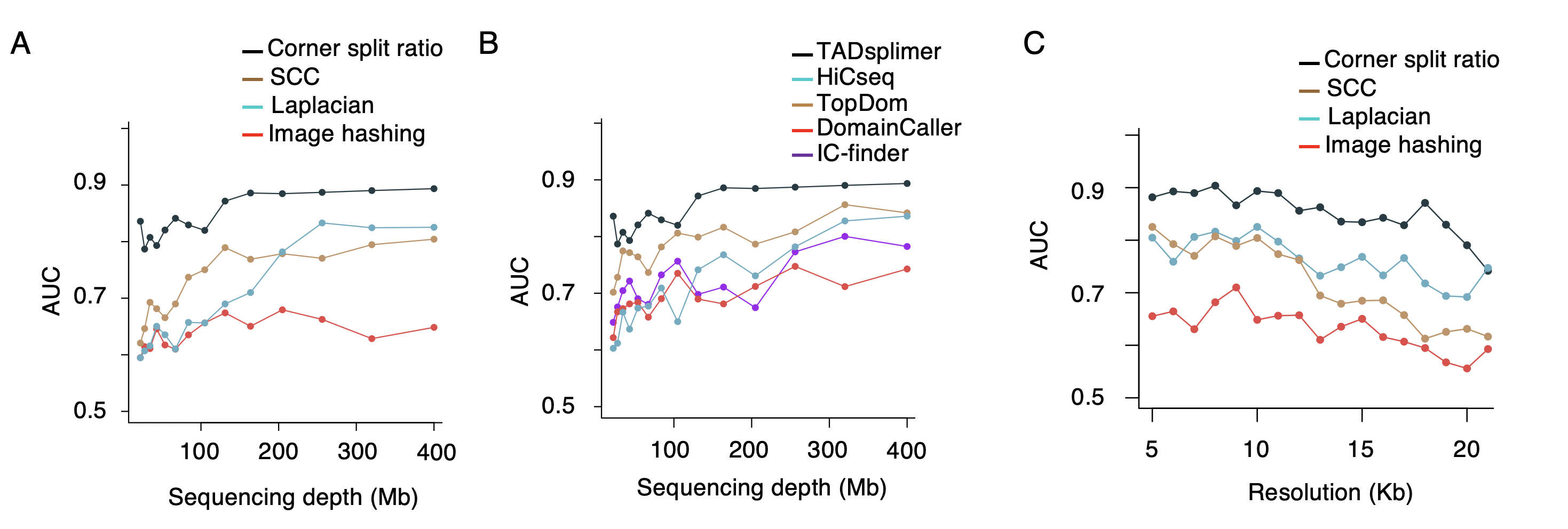


**Fig. S1 TADsplimer detect TAD splits and mergers robustly**

**(A)** AUC of ROC curve plotted against Hi-C sequencing depth to show performance of the four alternative methods in TADsplimer for scoring TAD splits. **(B)** AUC of ROC curve plotted against Hi-C sequencing depth to show influence of the five TAD identification methods on detection of TAD splits. **(C)** AUC plotted against Hi-C resolutions to show the performance of four alternative methods in TADsplimer for scoring TAD splits.

**
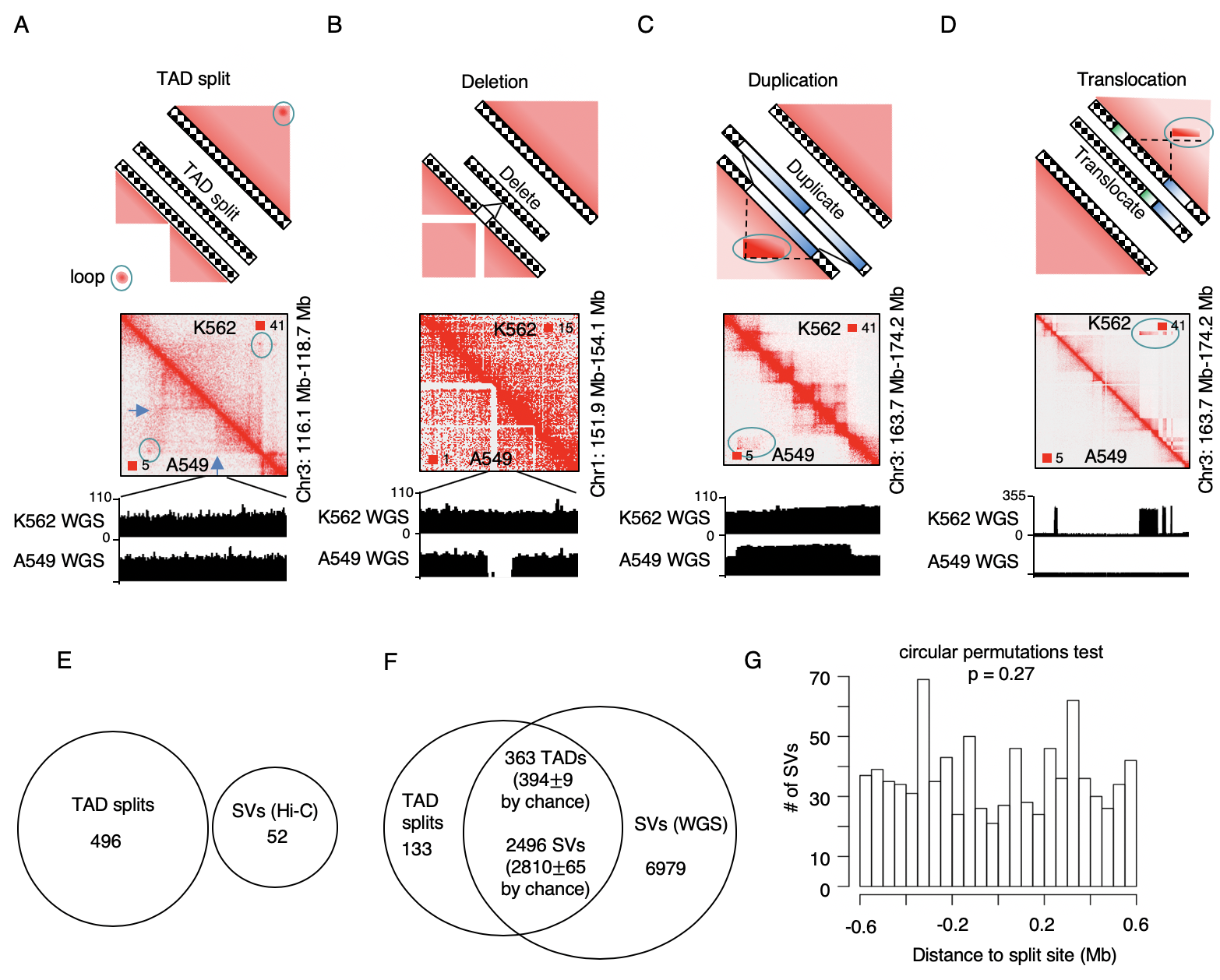
**

**Fig. S2 TAD split detected by TADsplimer between K562 and A549 cell lines can be independent of genetic structure variations**

**(A-D)** Heatmaps to show chromatin interactions expected in Hi-C data (top panels), observed in Hi-C data (middle panels), and whole genome sequencing signal (bottom panels) at TADs associated with TAD split (A), genomic deletion (B), genomic duplication (C), and genomic translocation (D). Blue circle indicate chromatin loops that were not disrupted by the TAD splits (A) or chromatin interactions altered by genetic structure variations (C-D) **(E)** Venn diagram to show the overlap between the TADs whose pattern changes are due to genetic structure variations and the TADs associated with splits or mergers. **(F)** Venn diagram to show the overlap between TADs associated with splits or mergers and genetic structure variations defined by whole genome sequencing data. **(G)** Distribution of genetic structure variations around split sites of TADs.

**
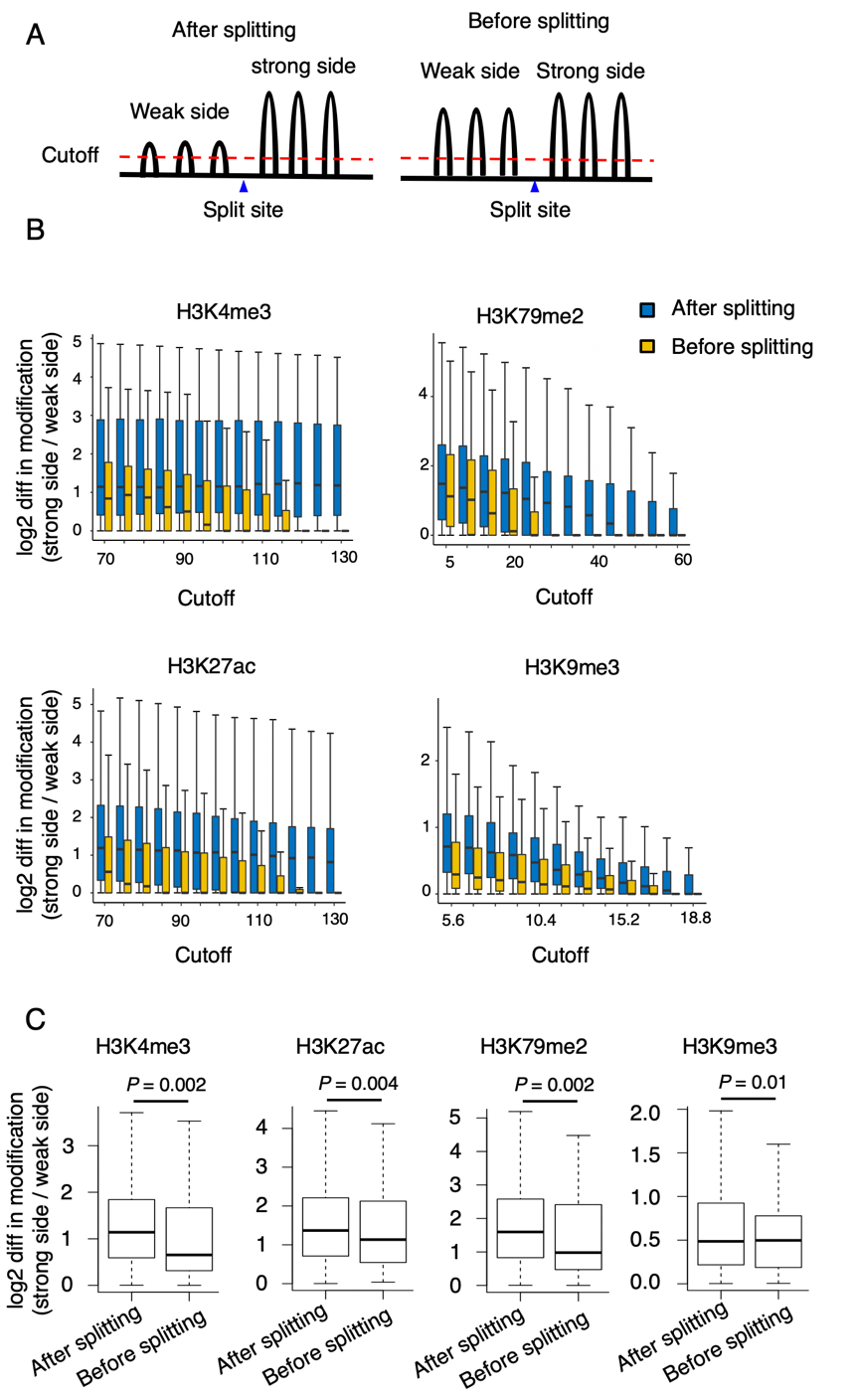
**

**Fig. S3 TADs splits and mergers are associated with change of chromatin stages at one side of the split sites**

**(A)** Cartoons to show histone modification signal in TAD after and before splitting. **(B)** Boxplot to show difference in histone modification signals between the two sides of split site. Histone modification signals are calculated only in the enriched peaks defined using individual cutoffs. (**C**) Boxplot to show difference in absolute ChIP-Seq read counts for histone modifications between the two sides of split site. P value were calculated by Wilcox test.
