# Supplementary material for: TADsplimer reveals splits and mergers of topologically associating domains for epigenetic regulation of transcription
Source: Genome Biol. 2020 Apr 2;21:84. doi: 10.1186/s13059-020-01992-7 (PMC7114812; doi:10.1186/s13059-020-01992-7)
Supplement: Supplementary file 2 — Additional file 2: Review history. [file 13059_2020_1992_MOESM2_ESM.docx]

Review History

**First round of review**

**Reviewer 1**

**Were you able to assess all statistics in the manuscript, including the appropriateness of statistical tests used?**

No

**Were you able to directly test the methods?**

No

**Comments to author:**

In the manuscript GBIO-D-19-01105, Wang et al presented TADsplimer, a bioinformatic tool for identifying TAD splits and fusions between different cell types. By applying TADsplimer to a diversity of Hi-C datasets, the authors observed that TAD splitting and merging (i) occurred between IMR90 and HUVEC cells are biologically relevant; (ii) interestingly, are largely independent of genetic alternations; (iii) are associated with changes in the chromatin epigenetic state; and (iv) are associated with changes in RNA expression at one side of split site.

TADsplimer can potentially serve as a very useful tool for assessing the dynamics of TAD organization during development.

Specific concerns:

1)	As demonstrated by the authors, the performance of TADsplimer is dependent on the calling TADs. Hence, the accuracy and sensitivity of the identification of TAD splits and fusions by TADsplimer rely on its accuracy of TAD calling. It is important for the authors to demonstrate that the TAD calling of TADsplimer is as good as the other published TAD callers. It will be helpful if the authors can include the comparison results between TADsplimer and the four other TAD callers;

2)	In the manuscript, the authors didn't provide a way to validate their TADsplimer results. After TADs are called in each cell type, can one identify TAD splits, fusions and de novo TADs by simply comparing the TAD boundaries between two cell types? This might provide a way to assess the accuracy and sensitivity of TADsplimer;

3)	It is interesting but seems odd that the authors found that TAD splitting and merging are largely independent of chromosomal structure variations. Did the authors investigate what is the similarity of TAD organization between A549 and K562 cells? Did the structure variations cause any other type of TAD reorganization (e.g., de novo TAD formation, TAD position shifting, or TAD lost)?

**Reviewer 2**

**Were you able to assess all statistics in the manuscript, including the appropriateness of statistical tests used?**

Yes

**Were you able to directly test the methods?**

Yes

**Comments to author:**

Wang et al present a manuscript in which they describe a method for the identification of splits and mergers of TADs. This is an interesting subject, that is largely understudied and few tools exist to properly analyze this. After identifying the splits using their tool they associate them with epigenomic and transcriptomic data to characterize the importance. The method is interesting, but in the biological analyses the documentation was at points too sparse to properly assess what is going on which I have detailed below. Besides these points there are three main issues that should be resolved before publication.

TADsplimer can act as a TAD caller, however, there is no benchmarking of the tool. The authors should use replicate Hi-C dataset to estimate the reproducibility using Jaccard indices. For this the example of Forcato et al. 2017 Nat. Methods figure 3d can be followed. This should also be done for the splits and mergers analysis that is currently done on the simulated data. The authors should also calculate the Jaccard index of splits and mergers on replicate datasets. The authors have already analyzed the HUVEC replicates, so this should be no limitation. They should also use data from Rao et al. 2014 and Bonev et al 2017 for this, which also includes ample replicates. They should also overlay their TAD calls, merger/split calls with CTCF data to see if this is enriched (as one would expect).

Furthermore, the authors now compare IMR90 and HUVEC, but many more comparisons can be made when data from the Rao and Bonev papers are included. This could significantly strengthen their conclusions and the generalizability of their results.

Finally, the use of splits and mergers is ambiguous, because a split also indicates a merger and it just the point of view one takes. Only in time series experiments one can speak of splits or mergers. Therefore the authors should find a less ambiguous way of discussing this. This is also true for left-right terminology, which is also somewhat ambiguous. This is particularly important when the data is compared to other dataset, where it is often unclear how the analysis is done or which cell type is chosen. The authors should make more of an effort to make their analysis and figures more comprehensible.

General: is there any association with split sites and CTCF motif orientation? How does the algorithm perform on different resolutions (and what resolution is used? 5Kb?) What input is expected? Tsv matrices? Upper triangular or full, dense matrices?

Other points:

We were able to install the software and run it.

Figure 1A-D:

Indicate colourscale in Hi-C plots

Figure 1C

why is the HUVEC data more sparse in C than in A?

Figure 1J,K: Why is AUC not used as a measure?

Figure 2A:

Please use linear maps, rather than circular.

Figure 2B:

Please plot 6 aggregates: merge and split for both IMR90 and HUVEC (4) and for all TAD borders for IMR90 and HUVEC (2).

Figure 2C:

The result seems rather trivial, merged TADs are larger. Please use a permutation test to get more reasonable estimate of the p-value.

Figure 2D:

Genes/promoters are enriched at TAD boundaries, so finding more genes at split sites (which are also TAD boundaries) seems a trivial observation. How does the reported 3fold enrichment compare to 'stable' TAD boundaries. Also should compare to equidistantly distributed sites. Also missing x-axis unit (Mb?).

Figure 2E:

Enriched compared to what? Should compare to 'stable' TAD boundaries Is this the nominal p-value or corrected?

Figure 2F-G:

Should choose more contrasting colours for Laplacian/SSC and/or adjust the legend order to follow the order in the plot. It seems like the GO terms chosen were those identified with the Corner Split Ratio / TADsplimer; are these also the top GO terms identified with the other methods?

Figure S1C: Drawing does not match the Hi-C map, but also the explanation is incorrect.

Figure S1E:

If sets are disjoint, Venn/Euler diagram should show no overlap.

Figure S1F:

Are the SV randomly permuted or circularly permuted? Text says permutation happened 1000 times, please provide a measure of the spread.

Figure S1G:

Please perform circular permutation. What unit is the x-axis? Mb?

Figure 3B:

Y-axis should indicate that it is the absolute ratio. Should probably also compare to regular TAD boundaries and not just the polar opposites (split vs merge). Use permutation test to calculate the p-value.

Figure S2: What was defined as a histone modification site? What is 'signal'? Is the MACS2 cutoff used? How do the statistics hold up if absolute read counts at either side of the splitted TAD are used instead of just the peaks?

Figure 3D: How are split scores calculated? How were these score normalised?

Figure 4A: Expression is measured in what unit? Probably should use a log y-axis, since a large portion is near-zero. I don't understand the x-axis. Are these the same genes before and after split/merges or different genes during a merge or split? What number of genes are involved?

It should also be noted here that the effect size is practically zero.

Figure 4C: How many genes are involved per split site? How are individual genes taken into account? What percentage of changes in gene expression can be explained by TAD merges?

Textual points:

The text should be read and corrected by a native English speaker.

Please refrain from using the word "important" when there is no explanation why it is important.

p. 3 l. 23: There are only 2-fold fewer interaction between TADs than within TADs, therefore the statement that there are few interactions is incorrect.

p. 4. l. 38: circumscribed: wrong terminology

**Authors Response**

**Point-by-point responses to the reviewers’ comments:**

*Summary: We thank all reviewers for acknowledging the significance and novelty of our findings. For example:*

*•	“TADsplimer can potentially serve as a very useful tool for assessing the dynamics of TAD organization during development” (Reviewer #1)*

*•	“The method is interesting” (Reviewer #2)*

*•	“This is an interesting subject, that is largely understudied, and few tools exist to properly analyze this.” (Reviewer #2)*

*Further, all reviewers appeared to have read our manuscript carefully and provided many constructive suggestions. The major concerns include (a) Lack of comparison between TADsplimer and reported TAD-calling algorithms regarding their accuracy for TAD calling, which is part of the TADsplimer algorithm (Reviewers 1&2); (b) performance of TADsplimer was evaluated based on 2 sets of public data, should further evaluate the performance based on additional public datasets (Reviewer 2); (c) Can further evaluate TADsplimer using some additional reported statistical methods (Reviewer 2); (d) should further evaluate performance of TADsplimer at different Hi-C resolutions (Reviewer 2).*

*We have now satisfactorily addressed those concerns in the revised manuscript. Briefly:*

*1.	We utilized replicates of Hi-C data to evaluate algorithm performance for TAD calling (Figure S1E), as well as for detection of TAD splits and mergers (Figure S1F).*

*2.	We used 17 additional sets of Hi-C data from Rao et al. 2014, Bonev et al. 2017 and Zirkel et al. 2018 to evaluate algorithm performance (Figure S1E, F, G).*

*3.	We utilized additional statistical methods, e.g., AUC (Figure S1A, B, C) and Jaccard index (Figure S1E, F), that were frequently reported to be useful for evaluating performance of Hi-C related algorithms.*

*4.	We tested the performance of alternative algorithms at different Hi-C resolutions. The result indicated that our default algorithm of TADsplimer has been optimal and thus show the best performance at all tested resolution (Figure S1C),*

*With these analyses, and with the additional analyses in response to other minor concerns, we believe that the manuscript has been significantly strengthened. Please see below for our detailed point-by-point responses. We have also highlighted major revisions by red color in the new manuscript.*

Response to Reviewer #1:

In the manuscript GBIO-D-19-01105, Wang et al presented TADsplimer, a bioinformatic tool for identifying TAD splits and fusions between different cell types. By applying TADsplimer to a diversity of Hi-C datasets, the authors observed that TAD splitting and merging (i) occurred between IMR90 and HUVEC cells are biologically relevant; (ii) interestingly, are largely independent of genetic alternations; (iii) are associated with changes in the chromatin epigenetic state; and (iv) are associated with changes in RNA expression at one side of split site.

TADsplimer can potentially serve as a very useful tool for assessing the dynamics of TAD organization during development.

*Response: We appreciate the reviewer for reading our manuscript thoroughly and summarized our findings accurately, and for highlighting that “TADsplimer can potentially serve as a very useful tool for assessing the dynamics of TAD organization during development”.*

Specific concerns:

Comment 1: As demonstrated by the authors, the performance of TADsplimer is dependent on the calling TADs. Hence, the accuracy and sensitivity of the identification of TAD splits and fusions by TADsplimer rely on its accuracy of TAD calling. It is important for the authors to demonstrate that the TAD calling of TADsplimer is as good as the other published TAD callers. It will be helpful if the authors can include the comparison results between TADsplimer and the four other TAD callers;

*Response: Thank you for this important suggestion. Accordingly, we compared TADsplimer to the other four TAD callers, including HiCseq, TopDom, DomainCaller and IC-finder, to evaluate their performances for TAD identification. We used 21 sets of Hi-C data from Rao et al. 20141, Bonev et al. 20172 and Zirkel et al. 20183, as was also suggested by the Reviewer 2. For each TAD calling method, we calculated Jaccard index between TADs defined from each pair of Hi-C replicate datasets. The results indicated that TADsplimer, HiCseq and TopDom had similar performance, and performed better than DomainCaller and IC-fider (Figure S1E).*

Comment 2: In the manuscript, the authors didn't provide a way to validate their TADsplimer results. After TADs are called in each cell type, can one identify TAD splits, fusions and de novo TADs by simply comparing the TAD boundaries between two cell types? This might provide a way to assess the accuracy and sensitivity of TADsplimer;

*Response: Following this constructive suggestion, we identified TADs in a pair of simulated Hi-C datasets, and then tried to detect TAD splits by simply comparing the TAD boundaries from the two datasets (Figure R1A). We again used TADsplimer to detect TAD splits between this pair of simulated datasets. To measure the performance of these two methods, we calculated Jaccard index between the identified TAD split sites and the simulated TAD split sites. The results indicated that the performance of TADsplimer is significantly better than simply comparing the TAD boundaries (Figure R1B). This result is reasonable. For a given TAD indicated in the step V in Figure 1F, assuming that the Hi-C signals in the regions A', B', A'', and B'' have the same value 5.1, whereas the signal value is 5.1 for the region A and 4.9 for the region B due to technical variation. When the signal cutoff for TAD calling is 5, the A', A, and A'' will be defined as a single TAD, the B' and B'' will be defined as two TADs with boundary at the region B. By simply comparing the TAD boundaries between two datasets, the region B will be wrongly defined as a split site. However, by further defining TAD splits based on the signal difference between regions A and B, TADsplimer will not define the region B as a split site, because the difference between regions A and B is minor (5.1 vs 4.9).*

Comment 3: It is interesting but seems odd that the authors found that TAD splitting and merging are largely independent of chromosomal structure variations. Did the authors investigate what is the similarity of TAD organization between A549 and K562 cells? Did the structure variations cause any other type of TAD reorganization (e.g., de novo TAD formation, TAD position shifting, or TAD lost)?

*Response: We have the same feeling with the reviewer. At the beginning, we also feel interesting that the TAD splits and mergers are largely independent of chromosome structure variations. After reading recent publications including these from the labs of Drs. Feng Yue (Dixon et al. 2018)4 and Mario Nicodemi (Bianco et al, 2018)5, we start to understand that although structure variations did cause TAD reorganization, the patterns of TAD signal changes caused by structure variations are different from the pattern associated with TAD split (Figure S2A-D). We further manually inspected the 52 individual TADs associated with the structure variations defined by the lab of Dr. Yue based on Hi-C signal pattern in A549 and K562, and still did not observe the pattern associated with TAD split (Figure R2). To investigate the global similarity of TAD organization between A549 and K562, we calculated the Jaccard indices for individual TADs between the two cell lines. The results indicated that the indices are significantly smaller than indices between Hi-C replicates for the same cell types, but significantly larger than expected by chance (random control) (Figure R3).*

Response to Reviewer #2:

Wang et al present a manuscript in which they describe a method for the identification of splits and mergers of TADs. This is an interesting subject, that is largely understudied and few tools exist to properly analyze this. After identifying the splits using their tool they associate them with epigenomic and transcriptomic data to characterize the importance. The method is interesting, but in the biological analyses the documentation was at points too sparse to properly assess what is going on which I have detailed below. Besides these points there are three main issues that should be resolved before publication.

*Response: We are grateful to the reviewer for the positive comments including “This is an interesting subject, that is largely understudied and few tools exist to properly analyze this” and “The method is interesting”. We would like to further appreciate the reviewer for the intriguing questions and constructive suggestions, which have led to a significant improvement of this manuscript as described in detail below.*

Comment 1.1: TADsplimer can act as a TAD caller, however, there is no benchmarking of the tool. The authors should use replicate Hi-C dataset to estimate the reproducibility using Jaccard indices. For this the example of Forcato et al. 2017 Nat. Methods figure 3d can be followed. This should also be done for the splits and mergers analysis that is currently done on the simulated data.

*Response: Thank you for this great suggestion. We downloaded the 21 datasets from Rao et al. 2014, Bonev et al. 2017, and Zirkel et al. 2018. To follow the Forcato et al. 2017 figure 3d, we calculated Jaccard indices of identified TADs between replicates. We compare the TAD calling algorithm of TADsplimer with the other TAD calling methods based on their associated Jaccard indices (Figure S1E). The results indicated that TADsplimer had similar performance when compared to HiCseq and TopDom, and had better performance when compared to DomainCaller and IC-fider.*

Comment 1.2: The authors should also calculate the Jaccard index of splits and mergers on replicate datasets. The authors have already analyzed the HUVEC replicates, so this should be no limitation. They should also use data from Rao et al. 2014 and Bonev et al 2017 for this, which also includes sample replicates.

*Response: Using the 21 datasets from Rao et al. 2014, Bonev et al. 2017, and Zirkel et al. 2018, we further defined TAD splits and mergers between samples based on each possible combination of replicates, e.g., combination I: cell type A replicate 1 versus cell type B replicate 1, combination II: cell type A replicate 1 versus cell type B replicate 2 ... … We then calculated Jaccard index of the TAD splits between these combinations, e.g., between the combinations I and II. The results indicated that TADsplimer has the best performance, as indicated by the largest Jaccard indices, when compared to other alternative algorithms for detection of TAD splits and mergers (Figure S1F).*

Comment 1.3: They should also overlay their TAD calls, merger/split calls with CTCF data to see if this is enriched (as one would expect).

*Response: We compared the number of CTCF peaks at split sites before and after the splitting between IMR90 and HUVEC cells, and used all TAD boundaries from the two cells as the control. The result indicated that number of CTCF peaks at split sites significantly increased after TAD splitting (Figure 2E).*

Comment 2: Furthermore, the authors now compare IMR90 and HUVEC, but many more comparisons can be made when data from the Rao and Bonev papers are included. This could significantly strengthen their conclusions and the generalizability of their results.

*Response: Thank you for the suggestion. We used TADsplimer to compare Hi-C data between 8 human cell types from the Rao papers and 3 mouse cell types from the Bonev papers. We have performed additional evaluation of algorithm performances based on these data (Figure S1E, F). Intriguingly, we next clustered the cell types by the number of TAD splits and mergers between cell types, and observed that the cell types with close developmental relationship tended to cluster together (Figure S1G).*

Comment 3.1: Finally, the use of splits and mergers is ambiguous, because a split also indicates a merger and it just the point of view one takes. Only in time series experiments one can speak of splits or mergers. Therefore, the authors should find a less ambiguous way of discussing this.

*Response: Thank you for this important suggestion. We have accordingly revised the writings to specify the point of view when we talked about TAD split or merger. It is now always described in a style similar to “…TAD split or merger in the cell line K562 in comparison to the cell line A549…”. Therefore, it will be clear that a TAD split or merger was observed in which cell type and in comparison to which cell type.*

Comment 3.2: This is also true for left-right terminology, which is also somewhat ambiguous. This is particularly important when the data is compared to other dataset, where it is often unclear how the analysis is done, or which cell type is chosen. The authors should make more of an effort to make their analysis and figures more comprehensible.

*Response: The “left-right” terminology has been replaced by “strong signal side” and “weak signal side” as defined by ChIP-Seq signal, so that it is quantitatively defined, straightforward to understand, and easy for other researchers to do analysis in the same way in future.*

Comment 4.1: General: is there any association with split sites and CTCF motif orientation?

*Response: We calculated the percentage of convergent CTCF motifs between TAD split sites and their adjacent TAD boundaries, and compared it to the percentage for other adjacent TAD boundaries. The results indicated that the percentage of convergent CTCF is similar for TAD split sites and other boundaries (Figure R4).*

Comment 4.2: How does the algorithm perform on different resolutions (and what resolution is used? 5Kb?)

*Response: Our test indicated the default algorithm in TADsplimer always performed better than other alternative algorithms at different resolutions (Figure S1C). We currently set 10kb to be the default resolution because the accuracy is similar but computational cost will be larger at higher resolution. However, users of TADsplimer have the freedom to set a different resolution based on the specific needs of their projects.*

Comment 4.3: What input is expected? Tsv matrices? Upper triangular or full, dense matrices?

*Response: The algorithm takes the full TSV matrices as its input.*

Comment 5: We were able to install the software and run it.

*Response: We are glad to know that the reviewer is able to install the software and run it. We invited our High-Performance Computing Server administrator to test the installation. We further invited two other postdoctoral fellows in our lab to install TADsplimer on their MAC laptops. All three investigators have also successfully installed and run TADsplimer.*

Comment 6: Figure 1A-D (the revised Figure 1A-D): Indicate color scale in Hi-C plots

*Response: Thank you for the suggestion. We have indicated color scale in each Hi-C plot at the top right and bottom left corners in the revised figures (Figures 1A-D, 3A, 3C, S2A-D).*

Comment 7: Figure 1C (the revised Figure 1C): why is the HUVEC data sparser in C than in A?

*Response: Figure 1C shows a genome region which is 0.8 Mb, while the Figure 1A shows a genome region which is 4.6 Mb. Therefore, the HUVEC data appears to be sparser in C than in A.*

Comment 8: Figure 1J, K (the revised Figure 1J, K): Why is AUC not used as a measure?

*Response: We have added figures to further use AUC as a measure (Figure S1A, B), and similarly, still observed better performance for TADsplimer in comparison to other alternative algorithms.*

Comment 9: Figure 2A (the revised Figure 2A): Please use linear maps, rather than circular.

*Response: Thank you for this suggestion. We have changed the circular map to linear maps in Figure 2A.*

Comment 10: Figure 2B (the revised Figure 2B): Please plot 6 aggregates: merge and split for both IMR90 and HUVEC (4) and for all TAD borders for IMR90 and HUVEC (2).

*Response: We have added plots for all TAD borders from IMR90 and HUVEC cells (Figure 2B).*

Comment 11: Figure 2C (the revised Figure 2C): The result seems rather trivial, merged TADs are larger. Please use a permutation test to get more reasonable estimate of the p-value.

*Response: Thank you for the suggestion. We have used permutation test to estimate the p-value (Figure 2C). For a more meaningful comparison, we further added a plot to show sizes of the other TADs that were not defined to be split or merged. Intriguingly, sizes of these other TADs were significantly smaller than sizes of the TADs in the merged status, but were close to sizes of the TADs in the split status.*

Comment 12: Figure 2D (the revised Figure 2D): Genes/promoters are enriched at TAD boundaries, so finding more genes at split sites (which are also TAD boundaries) seems a trivial observation. How does the reported 3fold enrichment compare to 'stable' TAD boundaries? Also, should compare to equidistantly distributed sites. Also missing x-axis unit (Mb?).

*Response: Thank you for the suggestion. We counted gene number at the same number of randomly picked boundaries that were not associated with splits or mergers, and repeated this random experiment for 1000 times. The results indicated no significant difference in gene enrichment between TAD split sites and “stable” TAD boundaries (Figure 2D, S1D). Genes are similarly enriched at TAD boundaries, no matter the boundaries are TAD split sites or not. We feel sorry for the confusion regarding x-axis unit. We have added the unit (Mb) and revised the label to indicate that the x-axis indicated normalized TAD size.*

Comment 13: Figure 2E (the revised Figure 2F): Enriched compared to what? Should compare to 'stable' TAD boundaries. Is this the nominal p-value or corrected?

*Response: We used the 2863 genes in the split or merged TADs to analyze the enriched pathways. In the revised manuscript we have used the same number of ‘stable’ TADs as control and repeated this random experiment for 1000 times (Figure 2F). Due to the small number of enriched pathways, we didn’t further correct the p value and thus used nominal p-value, which is also widely used to analyze the enrichment of pathway for genes6-8. Further, the p-values of these pathways are significantly lower for the split or merged TADs than for the control TADs, suggesting that the significance is specific and not simply due to multiple comparisons.*

Comment 14: Figure 2F-G (the revised Figure 2F-G): Should choose more contrasting colours for Laplacian/SSC and/or adjust the legend order to follow the order in the plot. It seems like the GO terms chosen were those identified with the Corner Split Ratio / TADsplimer; are these also the top GO terms identified with the other methods?

*Response: Thank you for the suggestion. We changed the colors and adjusted the legend order to follow the order in the plot. Due to space limitation, we are not able to include all pathways in the figures, the GO terms in figures 2F-G were a few representative pathways. In the revised manuscript, to perform a more unbiased comparison, we used heatmap to show all GO terms identified by at least one of the four methods for scoring TAD splits, including the corner split ratio, SCC, Laplacian, or image hashing methods (Figure S1H). Results indicated that 96 of the 158 pathways show the strongest enrichment for the corner split ratio method. For the five TAD calling methods including TADsplimer, TopDom, HiCseq, DomainCaller, and IC-finder (Figure S1I), 96 of the 188 pathways showed the strongest enrichment for TADsplimer.*

Comment 15: Figure S1C (the revised Figure S2C): Drawing does not match the Hi-C map, but also the explanation is incorrect.

*Response: Thank you very much for pointing this out! To match the Hi-C map in the bottom panel of Figure S2C, we revised the position of dash lines from top right corner to the bottom left corner in the highly interacted region, which is marked by the blue circle on the top panel of the new Figure S2C.*

Comment 16: Figure S1E (the revised Figure S2E): If sets are disjoint, Venn/Euler diagram should show no overlap.

*Response: Thank you for this suggestion. We have revised this Venn/Euler diagram to show no overlap (Figure S2E).*

Comment 17: Figure S1F (the revised Figure S2F): Are the SV randomly permuted or circularly permuted? Text says permutation happened 1000 times, please provide a measure of the spread.

*Response: The SVs are randomly permuted. We have added the standard deviation value (Figure S2F).*

Comment 18: Figure S1G (the revised Figure S2G): Please perform circular permutation. What unit is the x-axis? Mb?

*Response: Thanks for the suggestion. We have used circular permutations to estimate the local correlation between SVs and split sites. The results show that the SVs are randomly located in the TADs (p = 0.27). We have revised the x-axis label to indicate unit (Mb) (Figure S2G).*

Comment 19: Figure 3B (the revised Figure 3B): Y-axis should indicate that it is the absolute ratio. Should probably also compare to regular TAD boundaries and not just the polar opposites (split vs merge). Use permutation test to calculate the p-value.

*Response: Thank you for the suggestion. We have revised the Y-axis accordingly. We added the regular TAD boundaries and have used the permutation test to estimate the p-value (Figure 3B).*

Comment 20: Figure S2 (the revised Figure S3): What was defined as a histone modification site? What is 'signal'? Is the MACS2 cutoff used? How do the statistics hold up if absolute read counts at either side of the splitted TAD are used instead of just the peaks?

*Response: We used Danpos2 9, 10 which is previously developed by us to define histone modification sites based on ChIP-Seq data. ‘Signal’ is the normalized ChIP-Seq read count at each base pair. In response to the reviewer’s question, we further used the absolute read counts (from both peak regions and non-peak regions) at each side of the TAD split sites to perform analysis. The result indicated that the difference in histone modification signal is still bigger after splitting relative to before splitting (Figure R5), however, the difference became more significant when we perform analysis using only signal in the ChIP-Seq peaks. This is reasonable, because the signal outside of the peaks would have more noise signals, which are not likely to be affected by TAD splitting.*

Comment 21: Figure 3D (the revised Figure 3D): How are split scores calculated? How were these scores normalized?

*Response: The split scores were calculated by the split ratio method by default in TADsplimer, which is a measurement for the decreasing of the normalized interaction in the up-corner region of a split TAD (detail in the Equation (10)). We used the z-score of split score to normalize the scores among different cell types.*

Comment 22: Figure 4A (the revised Figure 4A): Expression is measured in what unit? Probably should use a log y-axis, since a large portion is near-zero. I don't understand the x-axis. Are these the same genes before and after split/merges or different genes during a merge or split? What number of genes are involved? It should also be noted here that the effect size is practically zero.

*Response: We feel sorry for this confusion. Expression is measured by FPKM. We have indicated the unit in Figure 4A in the revised manuscript. Because the difference mainly happened on the expressed genes, so it has been easy to see the difference in the original FPKM values. Therefore, we did not further change the values to log scale, but would like to thank the review for this suggestion.*

*For the x-axis, we revised the terminology so that is could be easier to understand. In the analysis of each type of histone modification, e.g., H3K4me3, the two sides around a TAD split site is now defined strong H3K4me3 side and weak H3K4me3 signal side. Thereafter, we compared the expression of the same genes at each side before (noted as “merger”) and after (noted as “split”) TAD split. We have further indicated the number of genes for each analysis in the revised figure 4A.*

Comment 23: Figure 4C (the revised Figure 4C): How many genes are involved per split site? How are individual genes taken into account? What percentage of changes in gene expression can be explained by TAD merges?

*Response: The average number of genes in each split site is 4.2. For genes whose FPKM values are each larger than 1, we calculated the fold difference of expression for each possible pair of genes from the two sides of each split site. We identified 2514 genes with differential expression between HSPC and DP. About 10% (242/2514) of these genes are associated with TAD mergers or splits.*

Textual points:

Comment 24: The text should be read and corrected by a native English speaker.

*Response: Thanks for the suggestion. We have invited Dr. John P. Cooke, a native English speaker, to further carefully revise the manuscript.*

Comment 25: Please refrain from using the word "important" when there is no explanation why it is important.

*Response: We agree with this and have made revision accordingly. For instances, we made the following changes:*

*changed “TADsplimer is optimal for detecting the TAD splits and mergers associated with important pathways” to “TADsplimer is optimal for detecting the TAD splits and mergers associated with cell fate related pathways” on page 8,*

*changed “genes in the TADs that were split or merged are associated with important pathways defining cell fate or function” to “genes in the TADs that were split or merged are associated with the pathways defining cell fate or function” on page 9.*

Comment 26: p. 3 l. 23: There are only 2-fold fewer interaction between TADs than within TADs, therefore the statement that there are few interactions is incorrect.

*Response: Thank you for pointing this out. We have changed this statement accordingly from “few interactions” to “about 2-fold fewer interactions”.*

Comment 27: p. 4. l. 38: circumscribed: wrong terminology.

*Response: Thank you very much for pointing this out. We have changed “circumscribed” to “organized”.*

*Revision references:*

*1.	Rao SS, Huntley MH, Durand NC, Stamenova EK, Bochkov ID, Robinson JT, Sanborn AL, Machol I, Omer AD, Lander ES and Aiden EL. A 3D map of the human genome at kilobase resolution reveals principles of chromatin looping. Cell. 2014;159:1665-80.*

*2.	Bonev B, Mendelson Cohen N, Szabo Q, Fritsch L, Papadopoulos GL, Lubling Y, Xu X, Lv X, Hugnot JP, Tanay A and Cavalli G. Multiscale 3D Genome Rewiring during Mouse Neural Development. Cell. 2017;171:557-572 e24.*

*3.	Zirkel A, Nikolic M, Sofiadis K, Mallm JP, Brackley CA, Gothe H, Drechsel O, Becker C, Altmuller J, Josipovic N, Georgomanolis T, Brant L, Franzen J, Koker M, Gusmao EG, Costa IG, Ullrich RT, Wagner W, Roukos V, Nurnberg P, Marenduzzo D, Rippe K and Papantonis A. HMGB2 Loss upon Senescence Entry Disrupts Genomic Organization and Induces CTCF Clustering across Cell Types. Molecular cell. 2018;70:730-+.*

*4.	Dixon JR, Xu J, Dileep V, Zhan Y, Songs F, Le VT, Yardimci GA, Chakraborty A, Bann DV, Wang YL, Clark R, Zhang LJ, Yang HB, Liu TT, Iyyanki S, An L, Pool C, Sasaki T, Rivera-Mulia JC, Ozadam H, Lajoie BR, Kaul R, Buckley M, Lee K, Diegell M, Pezic D, Ernst C, Hadjur S, Odom DT, Stamatoyannopoulos JA, Broach JR, Hardison RC, Ay F, Noble WS, Dekker J, Gilbert DM and Yue F. Integrative detection and analysis of structural variation in cancer genomes. Nat Genet. 2018;50:1388-+.*

*5.	Bianco S, Lupianez DG, Chiariello AM, Annunziatella C, Kraft K, Schopflin R, Wittler L, Andrey G, Vingron M, Pombo A, Mundlos S and Nicodemi M. Polymer physics predicts the effects of structural variants on chromatin architecture. Nat Genet. 2018;50:662-+.*

*6.	Damas J, Kim J, Farre M, Griffin DK and Larkin DM. Reconstruction of avian ancestral karyotypes reveals differences in the evolutionary history of macro- and microchromosomes. Genome Biol. 2018;19.*

*7.	Meddens CA, Harakalova M, van den Dungen NAM, Asl HF, Hijma HJ, Cuppen EPJG, Bjorkegren JLM, Asselbergs FW, Nieuwenhuis EES and Mokry M. Systematic analysis of chromatin interactions at disease associated loci links novel candidate genes to inflammatory bowel disease. Genome Biol. 2016;17.*

*8.	Barutcu AR, Lajoie BR, McCord RP, Tye CE, Hong D, Messier TL, Browne G, van Wijnen AJ, Lian JB, Stein JL, Dekker J, Imbalzano AN and Stein GS. Chromatin interaction analysis reveals changes in small chromosome and telomere clustering between epithelial and breast cancer cells. Genome Biol. 2015;16.*

*9.	Chen KF, Xi YX, Pan XW, Li ZY, Kaestner K, Tyler J, Dent S, He XW and Li W. DANPOS: Dynamic analysis of nucleosome position and occupancy by sequencing. Genome research. 2013;23:341-351.*

*10.	Chen K, Chen Z, Wu D, Zhang L, Lin X, Su J, Rodriguez B, Xi Y, Xia Z, Chen X, Shi X, Wang Q and Li W. Broad H3K4me3 is associated with increased transcription elongation and enhancer activity at tumor-suppressor genes. Nat Genet. 2015;47:1149-57.*

**Second round of review**

**Reviewer 1**

In the revised manuscript, the authors comprehensively addressed all the concerns raised by the reviewers in the previous round of review, and therefore this reviewer is happy to recommend "Accept".

**Reviewer 2**

The analysis method does not constitute a major advance over other methods.

Wang et al present a marginally improved version of their original manuscript. They have addressed my main criticism about the reproducibility of the TAD splits/mergers that are identified. However, there are still glaring mistakes in the statistical analysis. They also have not included answers to my questions in the manuscript so that the analyses can be repeated by others.

I feel there is a general lack of effort to explain what the authors have done or what is being measured in sufficient detail so that others can redo it. The authors provide a response to comment 4.3, 20, 21 and 23. However, this response is not included in the Methods section. These questions were crucial to understand the methodology.

Same is true for the color scales. It does not suffice to just include the number, if we cannot find anywhere in the manuscript what this number actually means.

If there is no difference for the enrichment of genes over split sites vs regular TADs, just remove this observation, as it does not lead to novel biological insight.

The GO analysis is flawed. If the p-value are not corrected for multiple hypothesis testing, there will always be some categories that will reach this nominal level of probability. I do not understand how a p-value of 1 can be reached in 1000 cases in the random analysis. Also p-values are not a quantitative measure, rather the enrichment score should be plotted. Better to leave out this analysis altogether.

Figure 2B: p < 0.16?

**Authors Response**

**Point-by-point responses to the reviewers’ comments:**

Point-by-Point Responses:

Reviewer #2: Wang et al present a marginally improved version of their original manuscript. They have addressed my main criticism about the reproducibility of the TAD splits/mergers that are identified. However, there are still glaring mistakes in the statistical analysis. They also have not included answers to my questions in the manuscript so that the analyses can be repeated by others.

*>>>Response: We thank the review for letting us know that we have addressed the reviewer’s main criticism about the reproducibility of the TAD splits/mergers that are identified. For the mistakes in the statistical analysis, we addressed them following the reviewer’s comments listed below. Please forgive us for answering to the reviewer’s questions but didn’t include some of the answers in the manuscript. It is because we failed to fully recognize the importance of these contents. We appreciate the reviewer for highlighting this, and accordingly, have included the answers in the new manuscript.*

1. I feel there is a general lack of effort to explain what the authors have done or what is being measured in sufficient detail so that others can redo it. The authors provide a response to comment 4.3, 20, 21 and 23. However, this response is not included in the Methods section. These questions were crucial to understand the methodology.

*>>>Response: Thank you for pointing out this problem. Following this instruction, to further make it easy for others to easily repeated our work, we have added more details of our analyses in the Methods section.*

*(1.1) In addition to the point-by-point responses to our reviewer’s comments (the points 1.2-1.5, 2, 3, and 4) described later, we have further performed these revisions:*

*(a) Added a “Data collection” section to describe the database accession number for each piece of genomic data, the version of each reference genome, and the version of each set of reference genes used in this analysis (between line 523 and line 544).*

*(b) Added an “Assessing the performance of TADsplimer” section to describe the workflow for assessing TADsplimer performance, including the version and parameters for each alternative algorithm or tool that were assessed (between line 580 and line 596).*

*(c) We further added a “Pathway enrichment analyses” section to describe the methods and packages that we used for each statistical test (between line 622 and line 636).*

*(d) We further added a “Statistical analyses” section to describe the methods and packages that we used for each statistical test (between line 638 and line 645).*

*(1.2) As point-by-point response to the previous comment 4.3, “What input is expected? Tsv matrices? Upper triangular or full, dense matrices?”, we added a section to describe input data format for TADsplimer between the line 568 and line 573 of the manuscript:*

*“A full Tab Separated Values (TSV) matrice at the resolution of 10 kb is used as the input for TADsplimer. In the real Hi-C data analysis of our study, this input data is the output contact map from the tool Juicer, with Vanilla coverage normalization at 10 Kb resolution. The row and column of the input matrice represent two genomic loci, and the value of an element in the input matrice indicates the frequency of interaction between the two genomic loci associated with the row and column.”*

*(1.3) For the previous comment 20, “Figure S2 (the revised Figure S3): What was defined as a histone modification site? What is 'signal'? Is the MACS2 cutoff used? How do the statistics hold up if absolute read counts at either side of the splitted TAD are used instead of just the peaks?”, we added a section to describe the workflow of ChIP-seq data analysis in the Methods section to explain how to define histone modification site between the line 599 and line 606:*

*“For ChIP-Seq data analysis, the sequencing reads from human cell lines were mapped to the human reference genome version hg19, and the sequencing reads of mouse cell lines were mapped to the mouse reference genome version mm9 using the bioinformatics tool Bowtie version 1.1.0 with default parameters. Only uniquely mapped reads were used for downstream analysis. We used the Danpos version 2.2.2 44,45 to define ChIP-Seq or DNase-Seq enrichment peaks. We normalized the average reads density across the genome to 0.65 reads/bp using Danpos2. The ‘Dpeak’ function in Danpos2 was used for peak calling with default parameters.”*

*For “what is ‘signal’”, we added the description between line 606 and line 607:*

*“In ChIP-seq data analysis, signal of ChIP-seq data is definded as the normalized ChIP-Seq read count at each base pair.”*

*For “How do the statistics hold up if absolute read counts at either side of the splitted TAD are used instead of just the peaks?”, we are sorry that we only putted this result to response letter but not added it to manuscript in the first round of review. In this round of review, we have added the results for the comparison of absolute read counts at either side of the split TADs in Figure S3C and in the manuscript between the line 270 and line 273:*

*“Furthermore, we used the absolute read counts (from both peak regions and non-peak regions) at each side of the TAD split sites to perform analysis. The result indicated that the difference in absolute read count is still bigger after splitting relative to before splitting (Figure S3C).”*

*(1.4) For the previous comment 21, “Figure 3D: How are split scores calculated? How were these scores normalized?”, we added a section to explain how the split score was calculated in the manuscript between line 573 and line 576:*

*“We used the corner split ratio method in TADsplimer to identify TADs splits or mergers with a cutoff value 0.45, which corresponds to a false positive rate (FDR) 0.01. All split scores used for further analysis were calculated by the corner split ratio method described above in detail.”*

*We further described how these scores were normalized between line 576 and line 578:*

*“We used the z-score method to further normalize the split score for comparison between the eight stages of T cell differentiation.”*

*(1.5) For the previous comment 23, “Figure 4C: How many genes are involved per split site? How are individual genes taken into account? What percentage of changes in gene expression can be explained by TAD merges?”, we added results for how many genes are involved per split site in line 327:*

*“The average number of genes in each split site is 4.2.”*

*We also added a section of RNA-seq data analysis in Methods to explain how the genes are taken into account between line 615 and line 620:*

*“To analyze the changes of RNA expression in the split or merged TADs between the eight stages of T cell differentiation, we first calculated the fold difference of FPKM value for each possible pair of genes from the two sides of each split site. We then analyzed whether this fold difference increase or decrease in response to TAD splits or mergers. Expressed genes, whose FPKM values are larger than 1, were included in the analysis”*

*For what percentage of changes in gene expression can be explained by TAD mergers, we described between line 352 and line 356:*

*“We further assessed what percentage of changes in gene expression can be explained by TAD split or mergers. We identified 2514 genes with differential expression between HSPC and DP. About 10% (242/2514) of these genes are associated with TAD mergers or splits.”*

2. Same is true for the color scales. It does not suffice to just include the number, if we cannot find anywhere in the manuscript what this number actually means.

*>>>Response: Thank you very much for this important suggestion. We are sorry for missing this important part of method. In the method section from line 546 and line 557, we now further added details for the processing of Hi-C data to generate Hi-C contact map and the visualization of Hi-C data:*

*“Processing of Hi-C data*

*All Hi-C data were processed using the bioinformatics toolkit Juicer Version 1.13.01 (https://github.com/aidenlab/juicer/wiki ) 41. We used the Juicer with default parameters to map Human Hi-C reads against the reference genome version hg19 to generate the .hic file, which is a compressed binary file that contains contact matrices, Similarly, mouse Hi-C reads were aligned against the reference genome version mm9 to generate the .hic files using Juicer with default parameters. We then used the Dump function in Juicer to extract .txt files, the matrix format of contact matrices, from the .hic files at the resolution of 10 kb with vanilla coverage normalization 12. Vanilla coverage normalization is a method in which the value of each element in a matrix is first divided by the sum of all values in the associated row and subsequently further divided by the sum of all values in the associated column.*

*Visualization of Hi-C data*

*Heatmaps of Hi-C matrices were visualized using the bioinformatics tool Juicebox (https://aidenlab.org/software.html ) 42 at the resolution of 10 kb using ‘Coverage’ normalization method, which applies vanilla coverage normalization to the Hi-C matrice. Color in each heatmap indicates the normalized number of Hi-C reads. Arc plot of Hi-C data was generated using the WashU Epigenome Browser (https://epigenomegateway.wustl.edu ).”*

3. If there is no difference for the enrichment of genes over split sites vs regular TADs, just remove this observation, as it does not lead to novel biological insight.

*>>>Response: Thank you very much for this important suggestion. We agree that it is better to remove the enrichment analysis of genes over split sites vs regular TADs. We have removed the enrichment analysis of genes in the revised manuscript.*

4. The GO analysis is flawed. If the p-value are not corrected for multiple hypothesis testing, there will always be some categories that will reach this nominal level of probability. I do not understand how a p-value of 1 can be reached in 1000 cases in the random analysis. Also, p-values are not a quantitative measure, rather the enrichment score should be plotted. Better to leave out this analysis altogether

*>>>Response: We appreciate the reviewer for further reminding us the importance of p-value correction. Using the commercial bioinformatics tool Ingenuity Pathways Analysis (IPA, Ingenuity System Inc, USA), we are now able to see enrichment of pathways with significant q value (B-H adjusted p value) (revised Figure 2H), e.g., pathways of vessel formation, which is a major function of endothelial cell, and other pathways such as the fibroblast cell proliferation, fibroblast movement, and cell death of fibroblast. To further show enrichment scores, we have indicated the number of observed genes for each pathway in the split / merged TADs, and the enrichment fold (comparing genes in the split / merged TADs with all genes in the reference gene set) (revised Figure 2H). We added this result to the revised manuscript between line 191 and line 212, and added a section to describe the methods of pathway enrichment analysis between line 622 and line 631.*

*Notably, thanks to the reviewers’ previous comments, we have further demonstrated the superior performance of TADsplimer in real Hi-C data based on Jaccard index analysis between replicates (Figure 2F, G). Analyses on real Hi-C, ChIP-Seq, DNase-Seq, and RNA-seq data also demonstrated relations between TAD splits or mergers and changes of histone modifications (Figure 3A, B, E), chromatin openness (Figure 3C, D, F), and gene expression (Figure 4A-C). With these important results, together with the results from the pathway analysis, we hope that we have successfully convinced our reviewer that our novel bioinformatics tool TADsplimer will allow users to reveal novel biological insights in many future researches.*

5. Figure 2B: p < 0.16?

*>>>Response: We are sorry for this typo. It should be “p=0.16” and we have corrected it in the figure.*

**Third round of review**

**Reviewer 2**

The authors have addressed my concerns about the lack of description of how the research was done.

The authors have replaced the original GO analysis with Ingenuity Pathway Analysis, which is an often used tool. However, due to its closed source and commercial nature I would suggest not using it in the future. The authors have once again plotted the p-values as a quantitative measure. Although this is standard practice it is not correct to use p-values quantitatively.
